# BST1 regulates nicotinamide riboside metabolism via its glycohydrolase and base-exchange activities

**DOI:** 10.1038/s41467-021-27080-3

**Published:** 2021-11-19

**Authors:** Keisuke Yaku, Sailesh Palikhe, Hironori Izumi, Tomoyuki Yoshida, Keisuke Hikosaka, Faisal Hayat, Mariam Karim, Tooba Iqbal, Yasuhito Nitta, Atsushi Sato, Marie E. Migaud, Katsuhiko Ishihara, Hisashi Mori, Takashi Nakagawa

**Affiliations:** 1 Department of Molecular and Medical Pharmacology, Faculty of Medicine, University of Toyama, 2630 Sugitani, Toyama, Toyama 930-0194 Japan; 2 Department of Molecular Neuroscience, Faculty of Medicine, University of Toyama, 2630 Sugitani, Toyama, Toyama 930-0194 Japan; 3 Research Center for Idling Brain Science (RCIBS), University of Toyama, 2630 Sugitani, Toyama, Toyama 930-0194 Japan; 4 Mitchell Cancer Institute, Department of Pharmacology, University of South Alabama, 1660 Springhill Avenue, Mobile, AL 36693 USA; 5 School of Bioscience and Biotechnology, Tokyo University of Technology, 1404-1 Katakuramachi, Hachioji, Tokyo, 192-0982 Japan; 6 Department of Immunology and Molecular Genetics, Kawasaki Medical University, 577 Matsushima, Kurashiki, Okayama 701-0192 Japan; 7 Research Center for Pre-Disease Science, University of Toyama, 2630 Sugitani, Toyama, Toyama 930-0194 Japan

**Keywords:** Metabolomics, Endocrine system and metabolic diseases, Nutrition

## Abstract

Nicotinamide riboside (NR) is one of the orally bioavailable NAD^+^ precursors and has been demonstrated to exhibit beneficial effects against aging and aging-associated diseases. However, the metabolic pathway of NR in vivo is not yet fully understood. Here, we demonstrate that orally administered NR increases NAD^+^ level via two different pathways. In the early phase, NR was directly absorbed and contributed to NAD^+^ generation through the NR salvage pathway, while in the late phase, NR was hydrolyzed to nicotinamide (NAM) by bone marrow stromal cell antigen 1 (BST1), and was further metabolized by the gut microbiota to nicotinic acid, contributing to generate NAD^+^ through the Preiss–Handler pathway. Furthermore, we report BST1 has a base-exchange activity against both NR and nicotinic acid riboside (NAR) to generate NAR and NR, respectively, connecting amidated and deamidated pathways. Thus, we conclude that BST1 plays a dual role as glycohydrolase and base-exchange enzyme during oral NR supplementation.

## Abstract (web summary)

Nicotinamide riboside (NR) is a NAD + precursor exhibiting beneficial effects against aging. Here the authors demonstrate that orally administered NR increases NAD + levels in a diphasic manner and that bone marrow stromal cell antigen 1 plays a crucial role for NAD + synthesis from NR.

## Introduction

Nicotinamide adenine dinucleotide (NAD^+^) is an essential molecule present in all living cells. Several important metabolic pathways, including glycolysis, tricarboxylic acid cycle, and fatty acid oxidation, use NAD^+^ as a cofactor to catalyze redox reactions by transferring electrons between NAD^+^ (oxidized form of NAD) and NADH (reduced form of NAD)^1^. It is also used as a substrate by other cellular enzymes, such as sirtuin, poly (ADP-ribose) polymerase (PARP), and NAD^+^ glycohydrolases CD38 and SARM1, to regulate various fundamental cellular processes including DNA repair, energy metabolism, epigenetic regulation, stress responses, and neuronal axon homeostasis^2–6^. Recent studies have shown that NAD^+^ can also be used as a nucleotide analog in DNA ligation and mRNA capping mechanisms^7,8^. Therefore, NAD^+^ is a crucial molecule for maintaining cellular homeostasis under both physiological and pathological conditions. Depending on the type of tissue, mammalian cells can synthesize NAD^+^ from tryptophan (Trp), nicotinic acid (NA), and nicotinamide (NAM) in the de novo, Preiss–Handler, and salvage pathways, respectively (Supplementary Fig. 1)^9–12^. Nicotinamide riboside (NR), nicotinamide mononucleotide (NMN), and nicotinic acid riboside (NAR) are also reported to be NAD^+^ precursors in several organisms and cells^13–17^. These pathways can also be classified as amidated or deamidated pathways based on the presence or absence of an amide group in the precursor and intermediate molecules. The NAD^+^ levels in cells are determined by the relative rates of synthesis and degradation^18^. Studies have shown that NAD^+^ levels are decreased in various metabolic diseases and aging^16^. Reduced synthesis and increased degradation of NAD^+^ by PARP and CD38 have been suggested to contribute to the decline in NAD^+^ levels in these conditions^19,20^. In fact, the expression levels of nicotinamide phosphoribosyltransferase (Nampt), a major rate-limiting enzyme in the salvage pathway, have been shown to be decreased with age, while those of PARP and CD38 were increased^12,16,21–23^. Decreased NAD^+^ levels disturb cellular metabolism and stress responses by affecting the activities of NAD^+^-dependent and NAD^+^-utilizing enzymes, resulting in acceleration of aging^16,24,25^. Conversely, elevated NAD^+^ levels in tissue have been shown to have beneficial effects in both physiological and pathological conditions^26,27^. Various approaches, such as supplementation with NAD^+^ precursors, activation of NAD^+^ biosynthetic pathways, and inhibition of NAD^+^ degradation, have been used to increase NAD^+^ levels in tissue^16,19,28^. Oral administration of amidated NAD^+^ precursors, NAM, NR, and NMN, has been demonstrated to be as an effective and practical approach to elevate NAD^+^ levels in vivo. Of these NAD^+^ precursors, NR is the most widely studied NAD^+^ precursor to date. NR is safe in human, and is considered more efficient in raising NAD^+^ levels compared to NAM and NA^15^. Additionally, NR administration has been shown to have beneficial effects in different disease conditions in both preclinical and clinical settings^27,29,30^. It has been reported that NR generates NAD^+^ via the NR salvage pathway, in which NR kinase (NRK) phosphorylates NR to generate NMN, followed by the transfer of the adenyl moiety of ATP to NMN by nicotinamide mononucleotide adenylyltransferase (Nmnat) to generate NAD^+^^31,32^. Thus, NRK and Nmnat in the NR salvage pathway are considered as important enzymes for NAD^+^ synthesis from NR. However, a previous study found that orally administered NR unexpectedly elevated levels of nicotinic acid adenine dinucleotide (NAAD) and nicotinic acid mononucleotide (NAMN) in various tissues of mouse and human^15^. Although this study demonstrated that the increased NAAD and NAMN were produced from the orally administered NR, the specific metabolic pathways involved have not yet been elucidated. According to the current knowledge of NAD^+^ metabolic pathways, NAAD and NAMN are not formed during conversion of NR to NAD^+^. Recently, Shats et al.^33^ showed that orally administered NAM is deamidated into NA by the gut microbiota and contributes to the generation of deamidated NAD^+^ metabolites, including NAAD and NAMN. The converted NA can be absorbed from the large intestine and contribute to the generation of NAAD and NAMN in different organs via the Preiss–Handler pathway. They also demonstrated that gut microbiota is necessary for NAD^+^ production from NR. Thus, this study raises a question whether orally administered NR uses the conventional amidated pathway to elevate NAD^+^ levels. Additionally, how these deamidated NAD^+^ intermediates generated by gut microbiota contribute to NAD^+^ generation after NR oral administration is still unclear.

In the present study, we show that orally administered NR increases NAD^+^ levels by two different mechanisms. In the early phase, NR is directly absorbed from the small intestine and contributes to NAD^+^ generation through the NR salvage pathway. In the late phase, NR is hydrolyzed to NAM by bone marrow stromal cell antigen 1 (BST1), and further metabolized by the gut microbiota to yield NA, contributing to NAD^+^ generation in the liver. Furthermore, we have discovered that BST1 has a base-exchange activity between NR and nicotinic acid riboside (NAR) using NA and NAM. This base-exchange activity is important in the small intestine to bypass the deamidated NAD^+^ precursors into the amidated pathway following the accumulation of deamidated metabolites. Thus, we concluded that NR metabolism is more complicated than originally thought, and it is important to consider the role of BST1 in NR supplementation therapy to protect from aging and aging-related diseases.

## Results

### Oral NR administration exhibits diphasic replenishment of NAD^+^

**Fig. 1:**
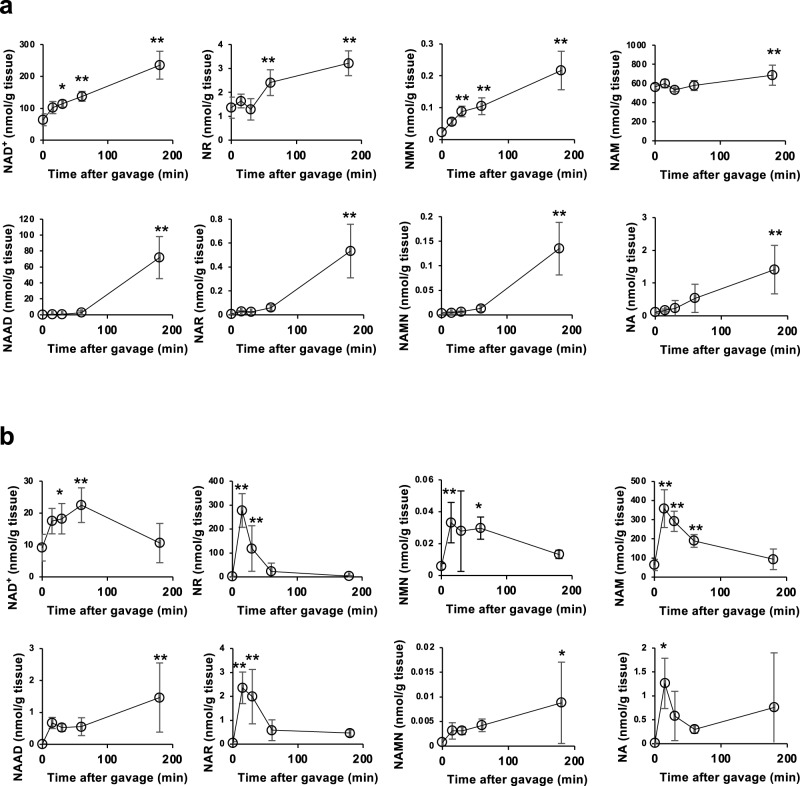
Time-course analysis of NAD^+^ metabolome after the gavage of NR. 400 mg/kg NR was administered to wild-type mice (*n* = 6 mice per time point) by gavage and killed after 0 m, 15 m, 30 m, 1 h, and 3 h for tissue collection. Relative abundance of NAD^+^ metabolome in the liver (**a**) and small intestine (**b**) were shown. Data are shown as mean ± S.D. *P < 0.05, **P < 0.01 as determined by one-way ANOVA followed by Tukey’s post-hoc tests. Source data are provided as a Source Data file.

It has been reported that NR supplementation resulted in a significant increase of deamidated NAD^+^ intermediates and that gut microbiota is involved in generating NAD^+^ and deamidated NAD^+^ intermediates^33^. To elucidate the NR metabolism pathway in vivo, NR was orally administered to C57BL/6N mice, and the NAD^+^ metabolome in the liver and small intestine was investigated at different time points between 0 and 3 h after administration. Although the levels of NAD^+^ in the liver gradually increased up to 3 h after administration (Fig. 1a), the levels in the small intestine were spiked at 1 h and dropped down to the basal level by 3 h after administration. Importantly, the levels of NR in the small intestine were profoundly elevated at 30 min and returned to near basal levels at 3 h, suggesting that NR was directly incorporated into intestinal cells and converted to NAD^+^ (Fig. 1b). Direct uptake of NR through the cellular membrane was also confirmed using cultured cells. As shown in Supplementary Fig. 2, NR levels were immediately increased 1 min after administration to culture media, while NAD^+^ levels increased a short time later. On the other hand, NR levels in the liver slightly increased at 15 min but returned to basal levels at 30 min. A more substantial rise in NR and NAD^+^ levels was observed at 3 h in the liver. Additionally, deamidated NAD^+^ metabolites, such as NAAD and NAMN, were significantly increased at this time, suggesting that the NAD^+^ synthesis from NR occurred at a later time point in the liver and was distinct from that at an earlier time point in the small intestine.

### Gut microbiota is involved in late phase NAD^+^ synthesis from NR

**Fig. 2:**
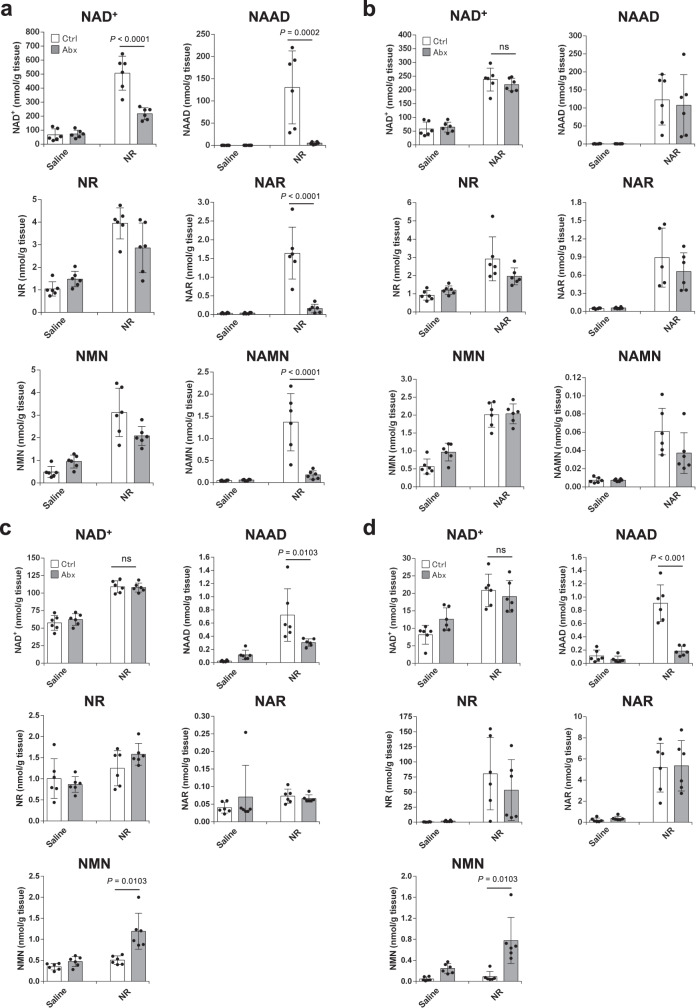
Gut microbiota is important for NAD^+^ increase in the late phase after NR administration. Wild-type mice were treated with either regular water (Ctrl) or antibiotic-containing water (Abx) for 3 days to deplete gut microbiota. They were then gavaged with 400 mg/kg NR or equivalent molar amount of NAR, and killed after 30 min or 3 h. **a**, **b** Concentrations of NAD^+^ metabolome in the liver were measured by LC-MS 3 h after the gavage of NR (**a**) (*n* = 6 mice per group) and NAR (**b**) (*n* = 6 mice per group). **c**, **d** Concentrations of NAD metabolome in the liver (**c**) (*n* = 6 mice per group) and small intestine (**d**) (*n* = 6 mice per group) were measured by LC/MS 30 min after the gavage of NR. Data are shown as mean ± S.D. ns not significant. Statistical significance was determined by one-way ANOVA followed by Tukey’s post-hoc tests. Source data are provided as a Source Data file.

A previous report suggested that gut microbiota contributed to the NAD^+^ synthesis from NR^33^. To confirm the role of gut microbiota in NR metabolism in the late phase, C57BL/6N mice were treated with cocktails of antibiotics for 3 days and then orally administered with NR. In agreement with the previous results using germ-free mice, pretreatment with antibiotics significantly suppressed the increase in NAD^+^ and deamidated NAD^+^ intermediates in the liver (Fig. 2a), while antibiotics pretreatment had no effect on the rise of NAD^+^ levels when mice were administered with NAR, a deamidated form of NR (Fig. 2b). We also confirmed that the increase in NAD^+^ levels by NA administration was not affected by pretreatment with antibiotics (Supplementary Fig. 3). Importantly, antibiotics pretreatment could not inhibit the NAD^+^ repletion in the liver and small intestine at the early phase (Fig. 2c, d). Taken together, we propose that NR metabolism in vivo is diphasic; (1) NR is directly incorporated and utilized in the small intestine in the early phase, and (2) gut microbiota-mediated deamidation is involved in the late phase of NAD^+^ synthesis from NR.

### NR is hydrolyzed to NAM in vivo

**Fig. 3:**
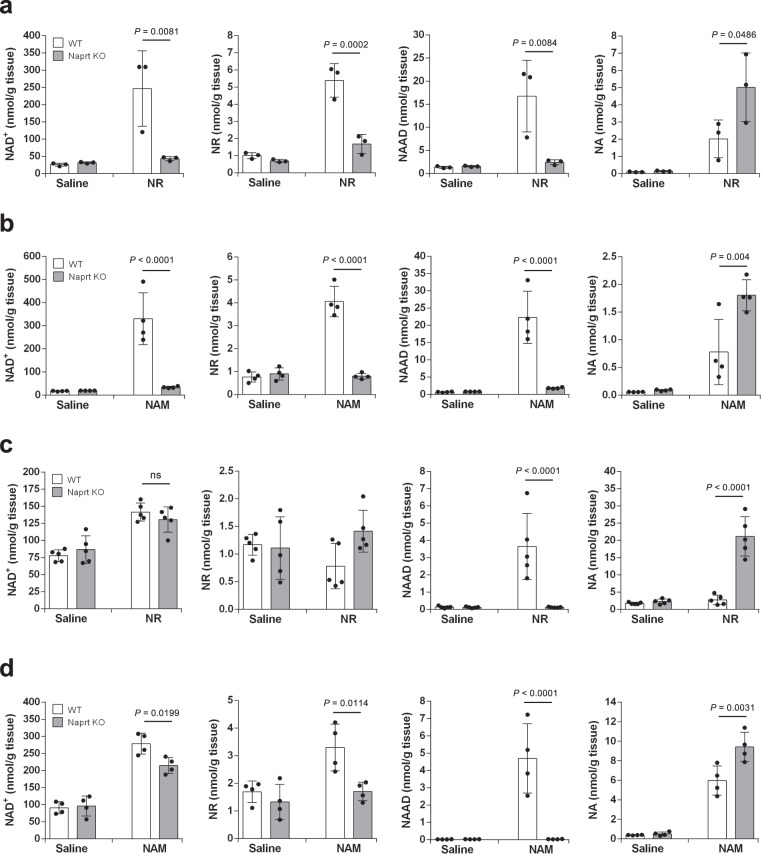
NR and NAM increased NAD^+^ levels in the liver of Naprt KO mice in the early phase but not in the late phase. Wild-type (WT) mice and Naprt KO mice were gavaged with 400 mg/kg NR or equivalent molar amount of NAM, then killed after 30 min or 3 h of the gavage. **a**, **b** Concentrations of NAD^+^ metabolome in the liver were measured by LC/MS 3 h after the gavage of NR (**a**) (*n* = 3 mice per group) or NAM (**b**) (*n* = 4 mice per group). **c**, **d** Concentrations of NAD^+^ metabolome in the liver were measured by LC/MS 30 min after the gavage of NR (**c**) (*n* = 5 mice per group) or NAM (**d**) (*n* = 4 mice per group). Data are shown as mean ± S.D. ns not significant. Statistical significance was determined by one-way ANOVA followed by Tukey’s post-hoc tests. Source data are provided as a Source Data file.

We have demonstrated that gut microbiota is necessary for NAD^+^ synthesis from NR in the liver; however, it is unknown precisely which NAD^+^ metabolic pathways are mediated by gut microbiota. When NR was administered to mice, the levels of both NA and NAR were increased in the liver after 3 h (Fig. 1a). It was previously reported that NAM is converted to NA by gut microbiota; thus, we presumed that the two possible pathways could either be: (1) NR is directly deamidated to NAR or (2) NR is hydrolyzed to NAM followed by the conversion to NA via microbiota-mediated deamidation. NA is converted to NAMN by nicotinic acid phosphoribosyltransferase (Naprt) in the Preiss–Handler pathway. To distinguish these possibilities, we orally administered NR to Naprt KO mice, in which NA cannot be utilized for NAD^+^ synthesis. First, we examined the NAD^+^ metabolism when NR was administered to Naprt KO mice. As shown in Fig. 3a, the increase in NAD^+^ and NAAD levels in the liver at 3 h was dramatically suppressed in NR-administered Naprt KO mice. We then orally administered NAM to Naprt KO mice and examined the NAD^+^ metabolome in the liver after 3 h (Fig. 3b). Similar to NR, the oral administration of NAM completely failed to increase NAD^+^ levels in the livers of Naprt KO mice at the late phase. In contrast, NR and NAM increased NAD^+^ levels in the liver at the early phase, regardless of the status of Naprt in mice (Fig. 3c, d). These results suggest that NR is degraded into NAM, and then converted into NA by gut microbiota in the late phase.

### BST1 catalyzes the conversion from NR to NAM

**Fig. 4:**
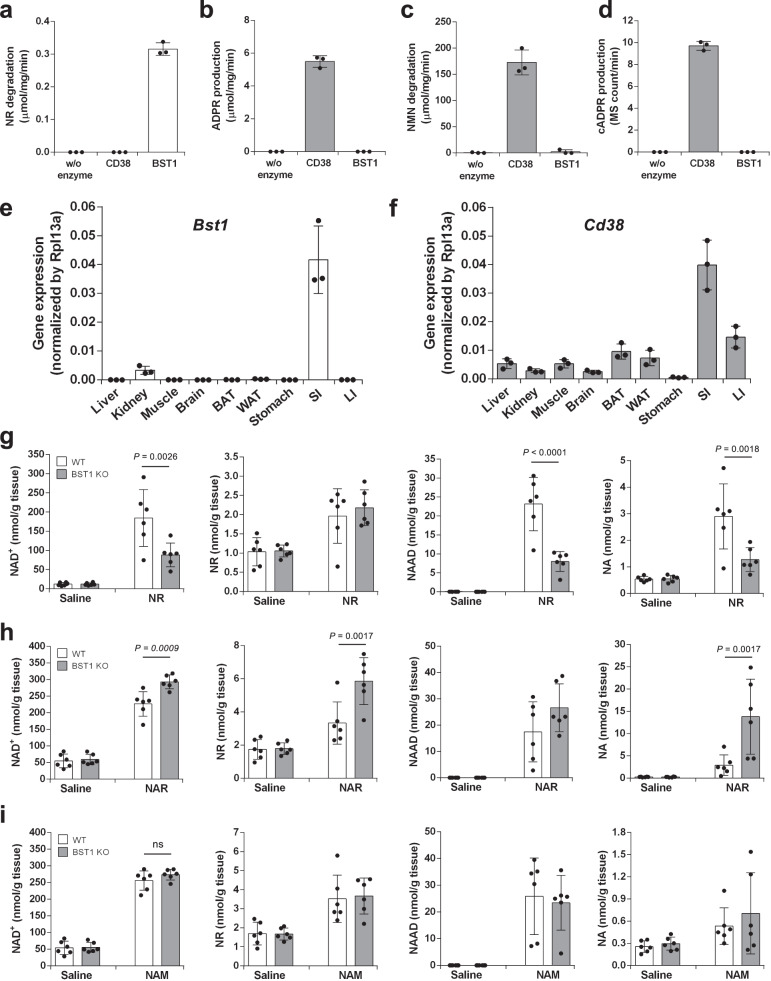
BST1 is required for the conversion of NR into NAM in vitro and in vivo. **a**–**d** Enzymatic activities of BST1 and CD38 as a glycohydrolase and cyclase were determined. **a** NR hydrolase activities were measured by the degradation rate of NR. (*n* = 3 independent experiments). **b** NAD^+^ hydrolase activities were measured by the production rate of ADP-ribose (ADPR). (*n* = 3 independent experiments). **c** NMN hydrolase activities were measured by the degradation rate of NMN. (*n* = 3 independent experiments). **d** ADP-ribosyl cyclase activities were measured by the production rate of cyclic ADP-ribose (cADPR). (*n* = 3 independent experiments). Data are shown as mean ± S.D. **e**, **f** mRNA expression of *Bst1* (**e**, *n* = 3 independent experiments) and *Cd38* (**f**, *n* = 3 independent experiments) in various tissues of wild type mice were measured by quantitative PCR (BAT brown adipose tissue, WAT white adipose tissue, SI small intestine, LI large intestine). Data are shown as mean ± S.D. **g**–**i** WT and BST1 KO mice were gavaged with 400 mg/kg NR, or equivalent molar amount of NAR or NAM, and then were killed after 3 h of the gavage. Concentrations of NAD^+^ metabolome in the liver were measured by LC/MS 3 h after the gavage of NR (**g**, *n* = 6 mice per group), NAR (**h**, *n* = 6 mice per group), and NAM (**i**, *n* = 6 mice per group). Data are shown as mean ± S.D. ns not significant. Statistical significance was determined by one-way ANOVA followed by Tukey’s post-hoc tests. Source data are provided as a Source Data file.

To generate NAD^+^ in the late phase, NR must first be converted to NAM. At this time, it is unclear which enzyme is responsible for the degradation of NR into NAM in vivo. It is well established that CD38 is a NAD^+^ glycohydrolase, which degrades NAD^+^ and NMN into NAM^34^. In mammals, CD38 has a paralog, BST1 (also known as CD157), which shares 25–30% amino acid sequence similarities^35^. Thus, we speculated that BST1 and/or CD38 may possess the activities required to degrade NR into NAM. Indeed, it was reported that BST1 exhibited an enzymatic activity to degrade NR and NAR in vitro^36^. To identify the enzyme that degrades NR into NAM, we examined the activity of BST1 and CD38 in the degradation of NAD^+^ metabolome in vitro. As reported previously, BST1 exhibited the required activity to degrade NR, but CD38 did not (Fig. 4a). On the other hand, activities of CD38 in the degradation of NAD^+^ and NMN were much higher than those of BST1 (Fig. 4b, c). We also observed the CD38 have glycohydrolase activity against nicotinamide adenine dinucleotide phosphate (NADP) and cyclase activity against NAD^+^, but BST1 did not (Supplementary Fig. 4 and Fig. 4d). These data suggested that CD38 and BST1 evolved differently and gained completely distinct substrate preferences. In particular, BST1 has a glycohydrolase activity specific to the nucleoside, NR, but CD38 has this activity for a broad range of nucleotides, including NAD^+^, NMN, and NADP.

### BST1 is necessary for the conversion of orally administered NR into NAD^+^

Assuming that BST1 is responsible for NR degradation into NAM, we analyzed the gene expression of *Bst1* and *Cd38* in various murine tissues. We found that *Bst1* was expressed mainly in the small intestine and slightly in the kidney (Fig. 4e), implying that BST1 degrades orally administered NR into NAM in the small intestine. As shown in Fig. 4f, *Cd38* is also expressed in intestinal tracts; therefore, CD38 may also contribute to the degradation of NAD^+^ and NMN in intestinal tract. To investigate the role of BST1 in NR metabolism in vivo, we orally administered NR to BST1 KO mice and examined the NAD^+^ metabolome. Although the deficiency of BST1 had no impact on NR levels in the liver (Fig. 4g), the increase in NAD^+^ levels upon NR administration was significantly diminished in BST1 KO mice. Similarly, the deletion of BST1 also suppressed the increase in NAAD and NA levels. On the other hand, the levels of NAD^+^, NR, and NA in BST1 KO mice were rather increased by NAR administration compared to those in WT mice (Fig. 4h). Furthermore, NAM administration to BST1 KO mice also increased NAD^+^ levels comparable to those in WT mice (Fig. 4i). These results suggest that BST1 contributes to the degradation of NR into NAM in the small intestine, while microbiota then converts NAM into NA in the large intestine.

### Alternative bypass route between the amidated and deamidated NAD^+^ synthesis pathways

**Fig. 5:**
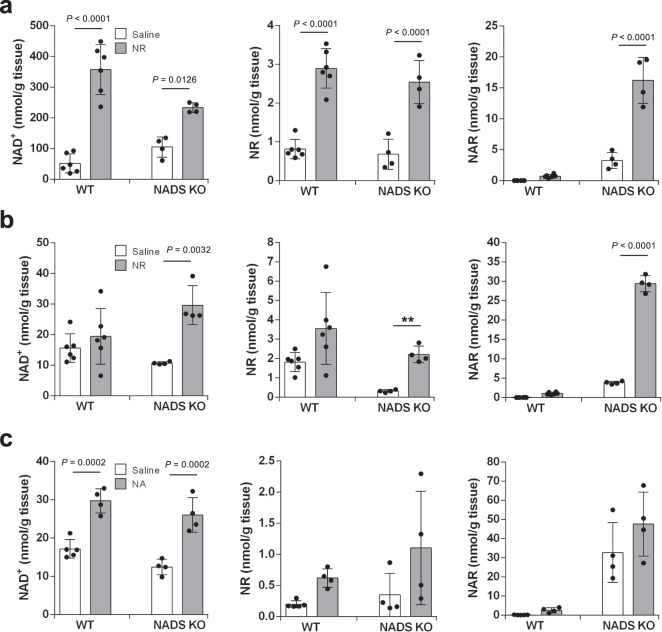
Oral administration of NR and NA increases NAD^+^ levels in the small intestine of NADS KO mice even in the late phase. Wild-type (WT) mice and NADS KO mice were gavaged with 400 mg/kg NR or equivalent molar amount of NA, and then were killed after 3 h of the gavage. **a**, **b** Concentrations of NAD^+^ metabolome in the liver (**a**, *n* = 5 mice per WT mice group, *n* = 6 mice per NADS KO mice group) and small intestine (**b**, *n* = 5 mice per WT mice group, *n* = 6 mice per NADS KO mice group were measured by LC/MS 3 h after the gavage of NR. **c** Concentrations of NAD^+^ metabolome in the small intestine was measured by LC/MS 3 h after the gavage of NA (*n* = 5 mice per saline-treated WT mice group, *n* = 4 mice per NA-treated WT mice group, and *n* = 4 mice per NA or saline-treated NADS KO mice group). Results were shown as mean ± S.D. Statistical significance was determined by one-way ANOVA followed by Tukey’s post-hoc tests. Source data are provided as a Source Data file.

NAD synthetase (NADS) is an enzyme that catalyzes the ATP-dependent amidation of NAAD to form NAD^+^ by transferring an amide group from l-glutamine^37^. Thus, it was expected that NADS KO mice would have similar NAD^+^ metabolism as Naprt KO mice when NR is orally administered. However, we found that the significant increase in NAD^+^ levels in the liver was observed in the late phase when NR was orally administered to NADS KO mice (Fig. 5a). In addition, we discovered that NR and NA could increase NAD^+^ levels in the small intestine of NADS KO mice to almost the same levels observed in WT mice (Fig. 5b, c). These results implied that a bypass route may exist between the amidated and deamidated NAD^+^ synthesis pathways downstream of Naprt.

### BST1 has a base-exchange activity against both NR and NAR

**Fig. 6:**
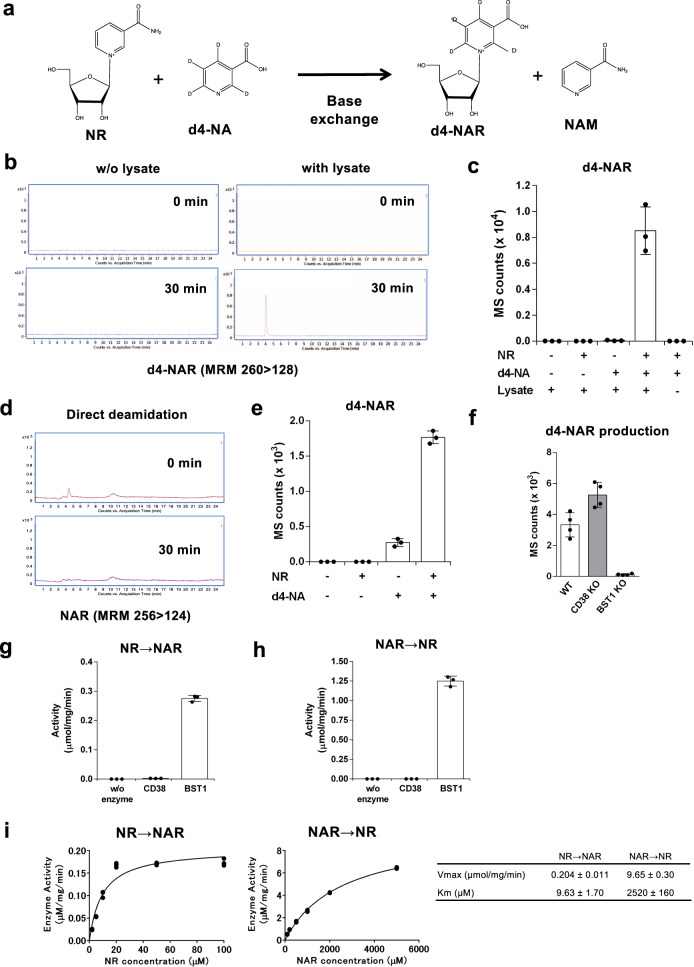
BST1 exhibited a base-exchange activity against NR and NAR. **a** Scheme showing a base-exchange reaction between non-labeled NR and d4-NA to generate d4-NAR and non-labeled NAM. **b**, **c** NR and d4-NA were incubated with mouse intestinal tissue lysate or buffer for 30 min at room temperature. Then, the whole mixture was subjected to LC/MS analysis to detect d4-NAR as a product of base-exchange reaction. Representative MRM chromatograms (**b**) and calculated MS counts (cof d4-NAR after the incubation with lysates were shown (*n* = 3 independent experiments). **d** Only NR was incubated with mouse intestinal tissue lysate for 30 min at room temperature. Then, the whole mixture was subjected to LC/MS analysis to detect non-labeled NAR as a product of direct deamidation. Representative MRM chromatograms of non-labeled after the incubation with lysates were shown. **e** Caco2 cells were cultured with non-labeled NR and d4-NA for 4 h. Then, metabolites were extracted from cells and subjected to LC/MS analysis for the detection of d4-NAR (*n* = 3 independent experiments). **f** NR and d4-NA were incubated with intestinal tissue lysates from WT, CD38 KO, or BST1 KO mice for 30 min at room temperature. Then, the mixture was subjected to LC/MS analysis for d4-NAR detection (*n* = 4 independent experiments). **g** Non-labeled NR and d4-NA were incubated with recombinant CD38 or BST1 protein for 30 min at room temperature. Then, the mixture was subjected to LC/MS analysis for the detection of d4-NAR (*n* = 3 independent experiments). **h** Non-labeled NAR and d4-NAM were incubated with recombinant CD38 or BST1 protein for 30 min at room temperature. Then, the mixture was subjected to LC/MS analysis for the detection of d4-NR (*n* = 3 independent experiments). **i** Enzyme activity of BST1 for NR and NAR to catalyze base-exchange reaction. Recombinant BST1 protein was incubated with various concentrations of NR and 1 mM NA or NAR and 1 mM NAM and the produced NAR or NR, respectively, was measured by LC/MS analysis. Michaelis–Menten curve fit was produced with GraphPad Prism software. Right panel shows *K*_m_ and *V*_max_. (*n* = 3 independent experiments) **c**, **e**–**h** Data are shown as mean ± S.D. **i** Each data point is shown. **b**, **d** Three independent experiments were performed. Source data are provided as a Source Data file.

Reportedly, CD38 catalyzes a base-exchange reaction to produce nicotinic acid adenine dinucleotide phosphate (NAADP) and NAM from NADP and NA^38,39^. It is also demonstrated that CD38 catalyzes a base-exchange reaction between NAAD and NAD^+^^40^. Thus, we speculated that BST1 may have a base-exchange activity against NR and/or NAR to connect the amidated and deamidated NAD^+^ synthesis pathways downstream of Naprt. To examine this possibility, we performed the base-exchange assays in vitro using murine tissue lysate. In this assay, deuterium-labeled NA (d4-NA) and non-labeled NR were incubated with tissue lysate and deuterium-labeled NAR (d4-NAR) was identified by LC/MS as a base-exchange product (Fig. 6a). As shown in Fig. 6b, c, an intense peak of d4-NAR appeared after the incubation with d4-NA and NR, suggesting the existence of base-exchange reaction from NR and NA to NAR and NAM in the tissue lysate. However, the tissue lysate incubated with only d4-NA could not produce d4-NAR, suggesting that d4-NAR was not converted from d4-NA by Naprt and 5′-nucleotidase. Additionally, the tissue lysate could produce neither d4-NAR nor non-labeled NAR without d4-NA, confirming that the tissue lysate had no direct deamidase activity (Fig. 6d). The existence of the base-exchange activity was also confirmed by using cultured cells (Fig. 6e). Importantly, tissue lysate from BST1 KO mice failed to produce d4-NAR in the presence of both NR and d4-NA (Fig. 6f), but that from CD38 KO mice rather exhibited the increased activity. To confirm whether BST1 solely catalyzed the base-exchange reaction, we incubated the substrates with recombinant BST1 (rBST1) or recombinant CD38 (rCD38). rBST1 could produce d4-NAR from d4-NA and NR, demonstrating that BST1 solely mediated the base-exchange reaction (Fig. 6g). rCD38 could also produce d4-NAR in vitro, but the activity was much less than rBST1 (Fig. 6g). It has been reported that acidic conditions are important for the base-exchange reaction by CD38^38^. Therefore, we investigated the activity of BST1-mediated base-exchange reaction in various pH. We found that BST1 exhibited the most robust activity at neutral pH though it still exhibited the obvious activity at acidic pH (Supplementary Fig. 5). Additionally, rBST1 could not use NAMN, NAAD, or NAADP as substrates for the base-exchange reaction even in acidic condition, while CD38 could use them as substrates but only at the acidic condition (Supplementary Fig. 6). We also examined whether rBST1 reversibly converted NAR and NAM to NR and NA by incubating rBST1 with non-labeled NAR and deuterium-labeled NAM (d4-NAM). Interestingly, rBST1 could produce deuterium-labeled NR (d4-NR), suggesting that the base-exchange reaction by BST1 is bidirectional (Fig. 6h). Furthermore, we analyzed the activity of BST1 against NR and NAR to catalyze their respective base-exchange reactions by Michaelis–Menten equation. We found that the *K*_m_ values of BST1 against NR and NAR were 9.63 ± 1.70 and 2520 ± 160 μM, respectively, while maximum enzyme velocity (*V*_max_) were 0.204 ± 0.011 and 9.65 ± 0.30 µmol/mg/min, respectively (Fig. 6i). Although BST1 has a higher affinity for NR than NAR as demonstrated by lower *K*_m_ value, the higher *V*_max_ for NAR indicates that NAR to NR reaction is much faster when there is a high concentration of NAR, as in the case of NADS KO mice. Taken together, BST1 appears to preferentially catalyze a base-exchange reaction from NR to NAR, however, it also catalyzes NAR to NR conversion at a high NAR concentration.

### Role of the base-exchange reaction against NR and NAR in vivo

**Fig. 7:**
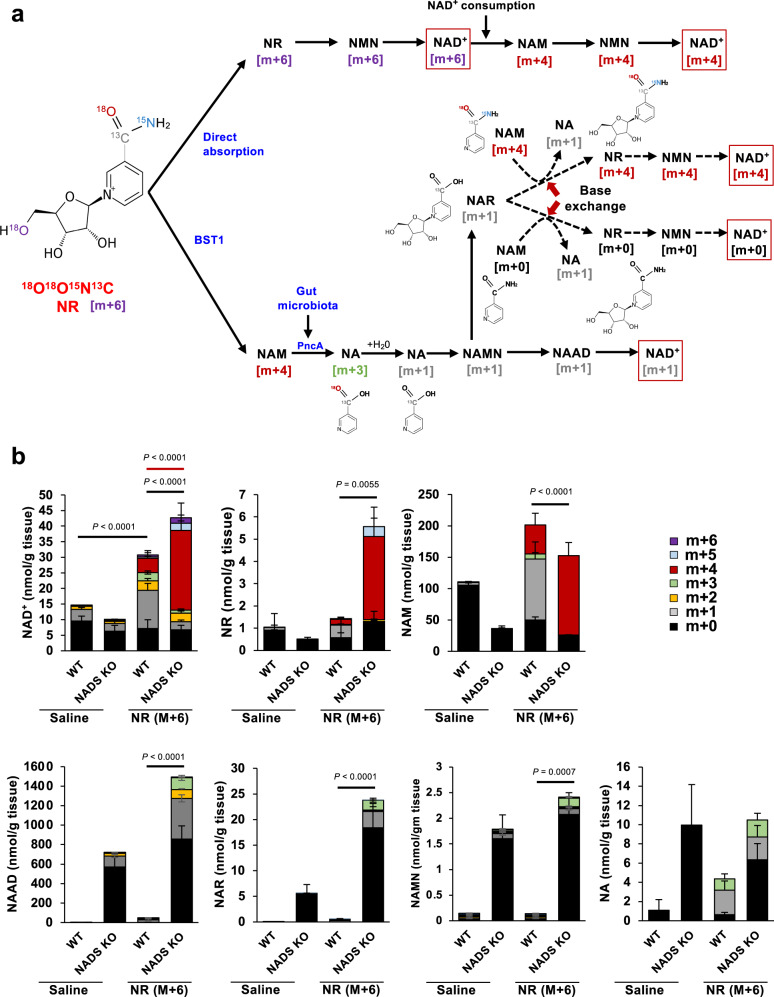
NAD^+^ is generated through distinct pathways in the small intestine of NADS KO mice during NR oral administration. Wild type (WT) and NADS KO mice were gavaged with quadruple-labeled NR, then were killed after 3 h of the gavage. **a** Scheme showing formation of isotope-labeled NAD^+^ metabolites after the oral administration of quadruple-labeled NR in the small intestine. **b** Concentration of unlabeled (m + 0) and labeled (m + 1, m + 2, m + 3, m + 4, m + 5 and m + 6) NAD^+^ metabolome in the small intestine was measured by LC/MS 3 h after the gavage of quadruple-labeled NR (*n* = 6 mice per group). Black and red lines indicated statistical analysis for m + 1 and m + 4 labeled metabolites, respectively. Data are shown as mean ± S.D. Statistical significance was determined by One-way ANOVA followed by Tukey’s post-hoc tests. Source data are provided as a Source Data file.

**Fig. 8:**
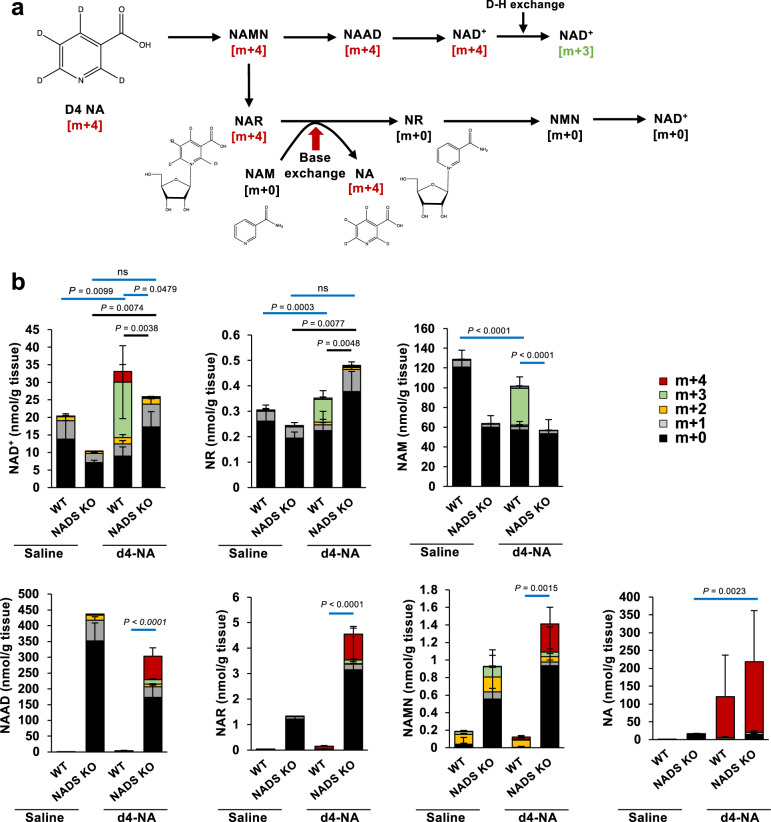
NAD^+^ is generated via base-exchange reaction in the small intestine of NADS KO mice. Wild-type (WT) and NADS KO mice were gavaged with d4-NA, then were killed after 3 h of the gavage. **a** Scheme showing formation of labeled and non-labeled NAD^+^ metabolome from d4-NA. **b** Concentration of unlabeled (m + 0) and labeled (m + 1, m + 2, m + 3, and m + 4) NAD^+^ metabolome in the small intestine was measured by LC/MS 3 h after the gavage of d4-NA (*n* = 6 mice per saline or d4-NA-treated WT mice group, *n* = 4 mice per saline-treated NADS KO mice group, *n* = 7 mice per d4-NA-treated NADS KO mice group). Data are shown as mean ± S.D. Black and blue lines indicated statistical analysis for non-labeled and labeled metabolites, respectively. ns not significant. Statistical significance was determined by one-way ANOVA followed by Tukey’s post-hoc tests. Source data are provided as a Source Data file.

In the late phase, orally administered NR is finally converted to NA in the digestive tracts through BST1 glycohydrolase activity and microbiota-mediated deamidation. NA produced from NR may be converted into NAD^+^ via the conventional Preiss–Handler pathway in cells. However, our identification of the base-exchange activity of BST1 highlighted the possibility that a base-exchange reaction against NR and NAR is involved in NR metabolism in vivo. Indeed, NAD^+^ levels in small intestine were fully increased after NR or NA administration in NADS KO mice compared to WT mice, suggesting that base-exchange reaction from NAR to NR occurs in NADS KO mice (Fig. 5b, c). To explore the role of BST1-mediated base-exchange reaction in the NR metabolism pathway, we administered [^18^O^18^O^15^N^13^C] NR ([m + 6] NR) (Fig. 7a), to WT and NADS KO mice and analyzed the NAD^+^ metabolome in the small intestine. This quadruple-labeled NR was purpose-made for this experiment, and the approach to its synthesis is entirely novel and extremely versatile (Supplementary Method). When [m + 6] NR is directly absorbed and contributes to NAD^+^ synthesis via the NR salvage pathway, [m + 6] and/or [m + 4] NAD^+^ will be produced (Fig. 7a). On the other hand, [m + 1] NAD^+^ will be generated when [m + 6] NR is degraded to NA via BST1 and microbiota followed by the conversion to NAD^+^ via Preiss–Handler pathway (Fig. 7a)^18^. As expected, [m + 6] NR administration to WT mice mainly produced [m + 1] NAD^+^ in the small intestine, suggesting that Preiss–Handler pathway mainly contributes to the NAD^+^ synthesis from NR in small intestine (Fig. 7b). On the other hand, [m + 6] NR administration to NADS KO mice largely increased [m + 4] NAD^+^ in the small intestine with no rise in [m + 1] and [m + 0] NAD^+^ (Fig. 7b). We initially hypothesized that a base-exchange reaction between [m + 1] NAR and [m + 0] NAM would occur in the small intestine of NADS KO mice to produce [m + 0] NR and then [m + 0] NAD^+^. However, [m + 0] NAD^+^ was not increased in the small intestine of NADS KO mice, and instead [m + 4] NAD^+^ was markedly increased accompanied by the rise of [m + 4] NAM and [m + 4] NR. We assume that [m + 4] NAD^+^ is also generated from [m + 4] NR, which was produced via the base-exchange reaction from [m + 1] NAR and [m + 4] NAM. The [m + 6] NR that we used in this study did not allow us to distinguish whether [m + 4] NAD^+^ was formed from the directly absorbed [m + 6] NR via the NR salvage pathway or via base-exchange reaction between [m + 1] NAR and [m + 4] NAM (Fig. 7a). Thus, to determine whether the base-exchange reaction contributes to NAD^+^ synthesis during NR administration, we administered d4-NA to NADS KO mice and analyzed the NAD^+^ metabolome in the small intestine. If d4-NA would be converted to NAD^+^ through the conventional Preiss–Handler pathway, the produced NAD^+^ would be labeled with deuterium in the pyridine ring of NAM ([m + 3] or [m + 4] NAD^+^) (Fig. 8a)^18^. On the other hand, if d4-NA detours around the reaction of NADS by using a base-exchange reaction from NAR to NR, labeled-NAR ([m + 4] NAR) would increase but NAD^+^ would not be labeled ([m + 0] NAD^+^) (Fig. 8a). As shown in Fig. 8b, d4-NA increased mainly [m + 3] NR and [m + 3] NAD^+^ in WT mice. On the other hand, d4-NA increased [m + 4] NAR, [m + 0] NR and [m + 0] NAD^+^ in NADS KO mice, suggesting that NAD^+^ is generated through the base-exchange reaction in NADS KO mice. These results indicated that NR-derived NA is mainly converted to NAD^+^ through the Preiss–Handler pathway, but the base-exchange reaction from NAR to NR may function as a backup route when the Preiss–Handler pathway is impaired.

### Preiss–Handler pathway is essential for NAD^+^ synthesis during chronic NR oral supplementation

**Fig. 9:**
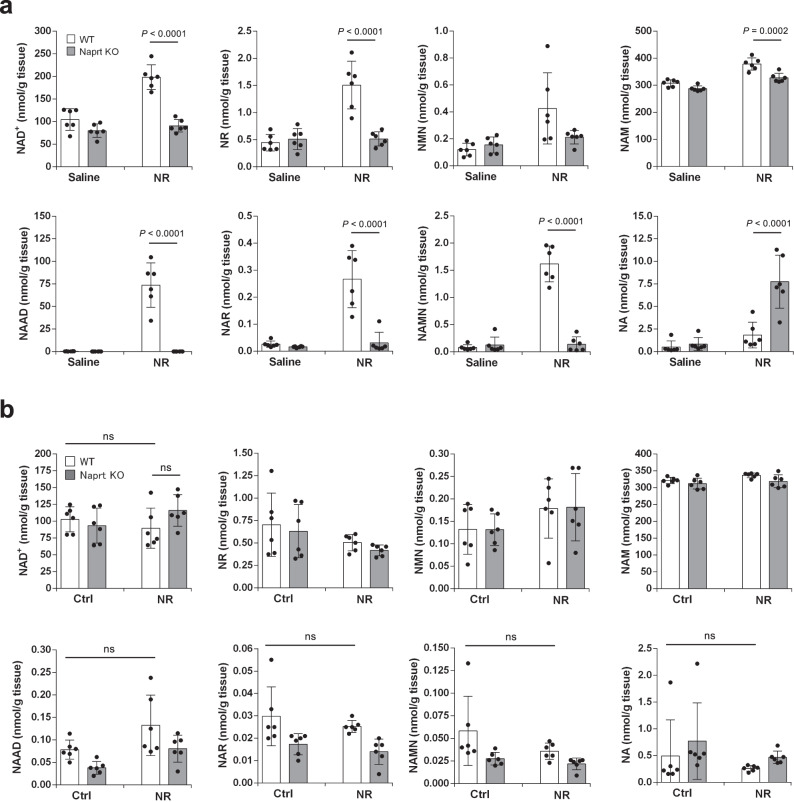
NAD^+^ is generated through Preiss–Handler pathway during chronic NR administration. Wild-type (WT) mice and Naprt KO mice were gavaged with 100 mg/kg NR or saline for 2 weeks or supplied NR in drinking water for 4 weeks. Mice were killed on last day after 3 h of gavage or in the morning for drinking water group. Concentrations of NAD^+^ metabolome in the liver were measured by LC/MS for gavage group (**a**) (*n* = 6 mice per group) and drinking water group (**b**) (*n* = 6 mice per group). Data are shown as mean ± S.D. ns not significant. Statistical significance was determined by one-way ANOVA followed by Tukey’s post-hoc tests. Source data are provided as a Source Data file.

Single-dose NR oral administration produced NAD^+^ in the liver mainly via the Preiss–Handler pathway (Fig. 3a). However, our data suggest that the direct absorption of NR has a small but significant contribution to NAD^+^ synthesis via the NR salvage pathway in the early phase (Fig. 3c). This raised a concern that the uptake of NR by the small intestine is overwhelmed at a high dose NR oral gavage (400 mg/kg/day), and NR may spill to large intestine to be converted into NA. Thus, we wished to ascertain whether direct NR absorption via the NR salvage pathway or the Preiss–Handler pathway play more important role in NAD^+^ synthesis during a low-dose chronic NR oral administration. For that we administered NR by oral gavage to WT and Naprt KO mice for 2 weeks at 100 mg/kg/day. Consistent with the single-dose NR oral administration, oral gavage for 2 weeks significantly increased NAD^+^ levels in the liver of WT mice, but failed to increase in Naprt KO mice (Fig. 9a). Significant increase of deamidated metabolites were observed in the liver of WT mice after 2 weeks of oral gavage, but not observed in that of Naprt KO mice. Finally, we administered NR by supplementation in drinking water to WT and Naprt KO mice for 4 weeks considering that, under this condition, the amount of NR that reaches the large intestine is limited, and NR may be directly absorbed mainly from the small intestine. However, compared to the gavage experiment, the supplementation of NR in drinking water could not increase NAD^+^ and deamidated metabolites in the liver of WT mice (Fig. 9b). Similarly, the rise of NAD^+^ and deamidated metabolites was not observed in Naprt KO mice. These results suggest that the Preiss–Handler pathway is still important even during the chronic NR oral administration.

## Discussion

**Fig. 10:**
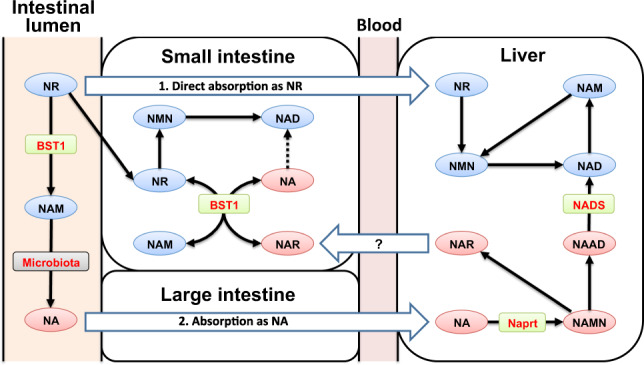
Schematic diagram showing metabolic fates of orally administered NR in vivo. Part of orally administered NR is directly absorbed from the small intestine. Orally administered NR is also degraded to NAM by BST1 in the small intestine. NAM derived from NR is further metabolized to NA by gut microbiota in the large intestine. Then, NA absorbed from the large intestine is used for the generation of NAD^+^ in the liver via the Preiss–Handler pathway. However, the base-exchange reaction for NR and NAR may function as a backup route when Preiss–Handler pathway is impaired.

The present study demonstrated that orally administered NR increased NAD^+^ levels in a diphasic manner (Fig. 10). First, in the early phase (within 1 h after administration), NR was directly taken up by the small intestine and utilized for NAD^+^ synthesis via the NR salvage pathway. In the late phase (~3 h after administration), NR increased NAD^+^ levels in a manner dependent on gut microbiota. Although the previous study demonstrated that gut microbiota was necessary for the conversion from NR to NAD^+^^33^, it was unclear whether NR was degraded into NAM or directly deamidated into NAR prior to the absorption by intestinal epithelial cells. Recently, it was also reported that orally administered NMN could be deamidated into NAMN by gut microbiota^41^. Here, we demonstrated that the increase in NAD^+^ levels in the liver was completely suppressed by a deficiency of Naprt in mice, suggesting that a large portion of NR was converted into NA before absorption and that gut microbiota could not directly deamidate NR into NAR. These data are in line with the recent observations by Shats et al. that the gut microbiota is important for late-phase NAD^+^ synthesis during NR oral administration^33^. Thus, we concluded that orally administered NR is degraded into NAM, which is subsequently deamidated into NA by the gut microbiota. Finally, NA derived from NR was converted into NAD^+^ through the Preiss–Handler pathway.

NAD^+^ was discovered as a coenzyme more than 100 years ago, and its metabolism has been thoroughly examined ever since. NAM, NA, and Trp are authentic, bioavailable NAD^+^ precursors through the de novo, Preiss–Handler, and salvage pathways, respectively^9–12^. Although various NAD^+^ synthesis pathways exist in tissues, NAD^+^ is primarily synthesized via the de novo pathway in liver and via the salvage pathway in non-hepatic tissues. However, when different precursors are available, tissues can utilize other pathways as well to synthesize NAD^+^. For instance, liver can use NA and NAM to synthesize NAD^+^ although the de novo pathway is the predominant pathway. It is, however, not clearly understood how these redundant pathways are regulated in different tissues. In 2004, NR was also identified as a novel NAD^+^ precursor in yeast and mammals^32^. The generation of NAD^+^ from NR can omit the step mediated by Nampt, a rate-limiting enzyme of the salvage pathway. Thus, NR may be considered a more preferable NAD^+^ precursor than others. Indeed, it has been demonstrated that supplementation with NR exhibits beneficial effects against aging and aging-related diseases, including obesity, diabetes, Alzheimer’s disease, mitochondrial myopathy, and disrupted circadian rhythms^42–48^. Additionally, NR has been shown to be orally bioavailable in humans without apparent adverse side effects^49,50^. Thus, numerous clinical studies have been conducted to examine its efficacy in preventing aging-related physical decline^49,51–53^. Our present study demonstrated that the rise of NAD^+^ levels in the early phase was significantly lesser than that in the late phase. This result suggested the direct conversion of NR into NAD^+^ through the NR salvage pathway is much less than was expected. We also confirmed that chronic NR gavage could also increase NAD^+^ levels in the liver of WT mice, but failed in Naprt KO mice. Thus, during chronic NR oral administration the Preiss–Handler pathway is important for NAD^+^ synthesis in liver. While low-dose NR supplementation in drinking water for 4 weeks failed to increase NAD^+^ as well as deamidated metabolites in the liver of WT mice. It may be due to the reason that mice consume water in small sips and the ingested NR with each sip was absorbed mainly from the small intestine and it did not contribute to NAD^+^ synthesis in liver. Compared to drinking water supplementation, oral gavage method closely resembles with the delivery of drugs in clinical treatments, such as tablets and capsules. Thus, considering this resemblance, we propose that the Preiss–Handler pathway is important during chronic NR oral administration in human. Indeed, NR supplementation in human by oral dosage forms has been shown to increase deamidated metabolites in peripheral blood mononuclear cells and blood^15,52^. Thus, the superiority of the NR conversion into NAD^+^ without Nampt is not anticipated when it is administered orally, and the effect of orally administered NR on NAD^+^ levels in the liver may be almost equivalent to that of NA itself. Thus, intravenous administration may be required to take full advantages of NR as a NAD^+^ precursor. Although the oral administration of NA often causes a side effect called niacin flush, a previous clinical study of oral administration of NR in humans showed that NR does not cause the apparent niacin flush^52^. In this regard, NR may be a better NAD^+^ precursor than NA.

BST1 is a paralog of CD38 and its contiguous gene duplicates on human chromosome 4 or murine chromosome 5^54–56^. CD38 reportedly has both NAD^+^ glycohydrolase and ADP-ribosyl cyclase activities. BST1 has also been reported to exhibit ADP-ribosyl cyclase activity to produce calcium mobilizing messengers, including cyclic ADP-ribose (cADPR)^57,58^. Thus, BST1 had been considered to possess similar activities as well, but the enzymatic activity of BST1 was apparently weaker than that of CD38^36,39,59^. Here, we demonstrated that BST1 has hydrolase activity specific to nucleoside, such as NR, but not to nucleotides, such as NMN and NAD^+^. Thus, our results suggest that BST1 evolutionally acquired the distinct substrate specificity from CD38. Furthermore, we discovered that BST1 has a base-exchange activity for NR and NAR using NA and NAM. This base-exchange activity of BST1 is distinct from that of CD38 where NADP was converted to NAADP in the presence of NA^36,39,59^. Consistent with this result, we also demonstrated that CD38 has a base-exchange activity for NADP to produce NAADP, but not BST1, indicating the unique base-exchange activity of BST1 for nucleosides. (Supplementary Fig. 6). Additionally, we have shown that oral administration of NR in NADS KO mice can increase the levels of NAD^+^ in the small intestine using this pathway. The oral administration of stable isotope-labeled NR and NA confirmed that the increase in NAD^+^ levels in the small intestine of NADS KO mice is due to a base-exchange reaction. Although the *K*_m_ value of BST1 for NAR is much higher that that for NR, we observed the conversion from NAR to NR in NADS KO mice. This base-exchange activity seems to be important in the small intestine to bypass the deamidated NAD^+^ precursors into the amidated pathway when deamidated metabolites, such as NAR, have accumulated. In this study, we only demonstrated that NR and NAR are substrates for BST1, but it is possible that BST1 also degrades or exchanges bases in other purine nucleosides, such as adenosine and guanosine.

Another group demonstrated that BST1 KO mice exhibited social deficits and anxiety-like behaviors, which were rescued by administration of NR^60^. In this study, NR administration increased NAD^+^ levels in the cortex and hypothalamus of BST1 KO mice. It is possible that the metabolic pathways of NR in these tissues are different from those in the liver and intestine. BST1 KO mice exhibit no apparent phenotype under normal conditions. It is important to reveal the physiological role of BST1-mediated NR degradation and base-exchange reaction in vivo. In particular, BST1 is highly expressed in the small intestine and immune cells. It has been reported that cADPR generated by BST1 in Paneth cells was necessary to mediate the beneficial effects of calorie restriction (CR) in the small intestine^61,62^. CR enhanced the self-renewal of intestinal stem cells (ISCs) through the suppression of mTOR signaling in Paneth cells in a manner dependent on BST1^61^. Another study also indicated that cADPR from Paneth cells activated ISCs through the upregulation of SIRT1 during CR^62^. These studies indicated the importance of cADPR in mediating the beneficial effects of CR. However, here we found that BST1 had no apparent cyclase activity to generate cADPR from NAD^+^. Thus, it is important to determine whether BST1 is necessary to mediate the effects of CR through its cyclase activity or through other enzymatic activities, such as glycohydrolase and newly discovered base-exchange activities.

Recently, it has been reported that the decline of NAD^+^ during aging was mediated by CD38 in pro-inflammatory macrophages^63,64^. Genotoxic and metabolic stresses promote cellular senescence followed by the induction of a senescence-associated secretory phenotype (SASP). SASP converts macrophages to an M1 phenotype, in which CD38 is expressed^63^. The decrease of NAD^+^ by CD38 occurred via the degradation of NMN by M1 macrophages^64^. We demonstrated that NAD^+^ and NMN were degraded by CD38, while NR was degraded by BST1. Thus, it is of further interest to determine whether BST1 is involved in aging and inflammation through the degradation of NR. In particular, BST1 is abundant in the intestine, and inflammatory bowel diseases are also mediated in part by macrophages. It will be interesting to examine roles of BST1 in these diseases.

The present study investigated the metabolic pathways of orally administered NR in mice. Although we have delineated the metabolic pathways of orally administered NR in the small intestine and liver, we did not investigate in other metabolically important organs like skeletal muscle, brain, kidneys, and heart. Future studies should consider combination approaches of using isotope-tracing methods with tissue-specific NAD^+^ enzymes knockout mice to fully understand NAD^+^ metabolism in other tissues during NR oral administration. We also acknowledge that we treated mice for a short time period of 2–4 weeks for chronic NR supplementation. Thus, longer treatment course would be essential to determine whether long-term NR oral supplementation can increase NAD^+^ levels independent of the Preiss–Handler pathway.

In conclusion, we determined the dual role of BST1 in the metabolic pathway of orally administered NR in vivo (Fig. 10). In particularly, the base-exchange activity for NR and NAR is a novel enzymatic activity of BST1. In addition, we elucidate the metabolism of orally administered NR in the small intestine and liver.

## Methods

### NAD^+^ precursors

NAD^+^, NADP, NAM, and NA were purchased from Nacalai Tesque (Kyoto, Japan). NAMN and NAAD were purchased from Sigma Aldrich (St Louis, MO, USA). NMN, NR, and NAR were provided from Mitsubishi Corporation Life Sciences Limited (Tokyo, Japan). Deuterium-labeled NAM (d4-NAM) and NA (d4-NA) were purchased from Toronto Research Chemicals (Toronto, Ontario, Canada) and CDN isotopes (Montreal, Quebec, Canada), respectively. Synthesis of quadruple-labeled [^18^O^18^O^15^N^13^C] NR is described in Supplemental Method.

### Animals

C57BL/6N mice were obtained from Japan SLC Inc. (Shizuoka, Japan). BST1 KO mice were obtained from RIKEN BRC (Stock No. RBRC02401)^65^. CD38 knockout mice were obtained from RIKEN BRC (Stock No. RBRC01462)^66^. NADS knockout mice were obtained from International Mouse Phenotyping Consortium (MMRRC:048728-UCD). Naprt knockout mice were generated using CRISPR-Cas9 techniques as described below. Animals were fed standard chow diet (CLEA Japan Inc., Japan) with free access to water. All the animals were kept under a controlled temperature and humidity (25 °C, 50%) with standard light condition (a 12:12 h light–dark cycle). All the animal experiments were approved by the Animal Experiment Committee at the University of Toyama and were performed in accordance with the Guidelines for the Care and Use of Laboratory Animals at the University of Toyama, which are based on international policies. All relevant ethical regulations have been complied.

### Generation of Naprt knockout mice

The injection and electroporation of guide RNA (gRNA) and Cas9 protein into C57BL/6N mouse zygotes was performed as previously described^67,68^. gRNA was designed to target exon 2 of mouse Naprt gene (NaprtE2: 5′-GCGTTCTTCGAGCACCTTCG-3′). Genotyping of offspring was performed by PCR using primers: (NaprtE2U1; 5′- CTGACCTCTGAGGGGACTTTTA-3′ and NaprtE2L1; 5′ CTCTTCCTAACACACCCAGCTC -3′) followed by DNA sequencing.

### NAD^+^ precursor treatment

For gavage experiments, 400 mg NR per kg body weight or equivalent mole amount of NAD^+^ precursors such as NA, NAM, and NAR were given to 8–10-week-old, male C57BL/6N mice. NAD^+^ precursors treated to animals were diluted in saline solutions and the same volume of saline solutions was used as a control. For drinking water experiments, NR was supplied in the drinking water as such mice consume 400 mg NR per kg body weight per day for 4 weeks. At the end of experiment, tissues were collected and immediately frozen in liquid nitrogen and kept at −80 °C until use.

### Antibiotics treatment

For antibiotics treatment, animals were treated with cocktails of vancomycin (0.5 g/L, LKT LAB, St. Paul, MN, USA), ampicillin (1 g/L,), metronidazole (1 g/L, Sigma-Aldrich), and neomycin (1 g/L, Sigma-Aldrich) for 3 days prior to the gavage experiments. At each time point, tissues were collected and immediately frozen in liquid nitrogen and kept at −80 °C until use.

### Cell culture

A549 and Caco2 cells (RIKEN BRC, Tsukuba, Japan) were cultured in Dulbecco’s Modified Eagle Medium supplemented with 10% fetal bovine serum at 37 °C under a gas phase of 95% air and 5% CO_2_. Cells were seeded at 2.5 × 105 cells per well on 12-well plate 1 day before harvest. For base-exchange reaction, NR and d4-NA were added at a concentration of 500 μM to Caco2 cells. After incubating for 4 h, cells were washed with the saline twice, and an ice-cold 50% methanol–50% water were added followed by the harvest using a cell scraper. For time course analysis, A549 cells were treated with NR at two different concentrations: 250 μM and 500 μM, and the cells were harvested after 0, 1, 5, 15, 60, and 240 min for NAD^+^ and NR measurements.

### NAD^+^ metabolomics

Metabolite extraction and NAD^+^ metabolomics were performed as previously described^69^. Tissues were grinded in an ice-cold 50% methanol–50% water at concentrations of 50 mg tissue/mL by using multi-beads shocker (Yasui Kikai, Japan) under optimal condition. Subsequently, 600 μl of lysate was mixed with 600 µl of chloroform and the mixture was vortexed for 10 s. The mixture was centrifuged at 13,000×*g* for 10 min at 4 °C. The upper phase (aqueous phase) was collected into a new tube and the same procedures were repeated. Then, the transferred aqueous phase was dried by using a SpeedVac SPD1010 (Thermo). Finally, the dried samples were reconstituted in LC/MS grade water (FUJIFILM Wako Pure Chemical Corporation, Osaka, Japan) and filtered with 0.45 μm Millex filter unit (Merck ltd. Tokyo, Japan) before the injection. Metabolites were analyzed by the Agilent 6460 Triple Quad mass spectrometer coupled with Agilent 1290 HPLC system. The system was operated by MassHunter Workstation-Data Acquisition (Version B.05.00, Agilent Technologies, Santa Clara, CA, USA). Analytes were separated by Atlantis T3 Column (2.1 × 150 mm, particle size 3 µm, Waters) using mobile phase A (5 mM ammonium formate) and mobile phase B (methanol) with a flow rate of 150 µl/min and a column temperature of 40 °C. The programmed mobile phase gradient was as following: 0–10 min, 0–70% B; 10–15 min, 70% B; and 15–20 min, 0% B. Detection of labeled NAD^+^ metabolites were performed by using modulated transitions of *m*/*z*, equal fragmentor voltage, and equal collision energy with non-labeled NAD^+^ metabolites. Data were analyzed by MassHunter Workstation-Quantitative Analysis (Version B.05.00, Agilent technologies, Santa Clara, CA, USA) and quantifications were performed by using the standard curve obtained from various concentrations of standard compounds. Individual chromatograms were extracted by MassHunter Workstation-Qualitative Analysis (Version B.05.00, Agilent technologies, Santa Clara, CA, USA).

### NAD^+^ glycohydrolase activity assay

Recombinant human BST1 and CD38 proteins were purchased from R&D systems (Minneapolis, MN, USA). rBST1 or rCD38 protein (1 ng for NAD^+^ and NMN degradation, 20 ng for NR degradation) were incubated with 0.1 mM NR, 0.2 mM NAD^+^, or 0.2 mM NMN in 10 μl of reaction buffer containing 25 mM Tris-HCl, pH 7.4 at 37 °C for 10 and 30 min. The reaction was stopped by adding 20 μl of 0.5 N perchloric acid. After a centrifugation, the supernatant was neutralized by adding the same volume of 1 M ammonium formate followed by dilution with LC/MS grade water and filtration using a 0.45 μm Millex filter unit for the detection by using LC/MS as described above.

### Base-exchange activity assay

For assays of base-exchange activity using murine tissues, small intestine from wild type, CD38 KO, and BST1 KO mice were grinded in 25 mM Tris-HCl, pH 7.4. After brief sonication, tissue homogenate was centrifuged at 13,000×*g* for 10 min at 4 °C. Supernatant was collected and protein concentration was determined by using Qbit assay (Thermo Fisher Scientific, Inc. Waltham, MA, USA). For the assay, 10 μg of tissue protein was incubated with 0.2 mM NR and/or 1 mM d4-NA in 10 μl of reaction buffer containing 25 mM Tris-HCl, pH 7.4 at 37 °C for 10 min. To check the base-exchange activities of rCD38 and rBST1, these proteins was incubated with 0.2 mM NR and/or 1 mM d4-NA in 10 μl of reaction buffer containing 25 mM Tris-HCl, pH 7.4 at 37 °C for 10 min. The reaction was stopped by adding 20 μl of 0.5 N perchloric acid. After centrifugation, the supernatant was neutralized by adding the same volume of 1 M ammonium formate followed by dilution with LC/MS grade water and filtration using a 0.45 μm Millex filter unit. For a reverse reaction of base-exchange assay, 0.2 mM NAR and 1 mM d4-NAM were used as substrates to produce d4-NR. d4-NAR and d4-NR were detected by LC/MS. For enzymatic activity assay of BST1, similar method as of the base-exchange reaction was used with various concentrations of NR (2, 5, 10, 20, 50, and 100 μM) and NAR (100, 200, 500, 1000, and 2000 μM).

### qPCR

Total RNAs were extracted from murine tissues by using TRI Reagent (Molecular Research Center, Inc., Cincinnati, OH, USA). cDNA was synthesized by using ReverTra Ace qPCR RT Master Mix with gDNA Remover (Toyobo, Osaka, Japan) according to the supplier’s protocol. Real-time PCR was performed by using THUNDERBIRD SYBR qPCR Mix (Toyobo) on Thermal Cycler Dice Real Time System II (Takara Bio). mRNA was quantified by Delta-Delta Ct method against Rpl13a as reference gene. The following primers were used for cDNA amplification: CD38, 5′-TCTCTAGGAAAGCCCAGATCG-3′ (F) and 5′-AGAAAAGTGCTTCGTGGTAGG-3′ (R); BST1, 5′-AGGGACAAGTCACTGTTCTGG-3′ (F) and 5′-AACTTTGCCATACAGCACGTC-3′ (R); Rpl13a, 5′-AGCGCCTCAAGGTGTTGGA-3′ (F) and 5′-GAGTGGCTGTCACTGCCTGGTA-3′ (R).

### Statistical analysis

Data are expressed means ± S.D. Data were analyzed using Graphpad Prism 9 software (version 9.1.2, GraphPad Software, San Diego, CA). The significant differences were evaluated by using one-way ANOVA followed by Tukey’s post-hoc tests. *P*-values <0.05 were determined as statistically significant.

### Reporting summary

Further information on research design is available in the Nature Research Reporting Summary linked to this article.

## Supplementary information

Supplementary Information

Reporting Summary

## Data Availability

The raw data generated for all figures (Figs. 1–10 and Supplementary Figs. 1–6) of this study are provided in the Source data file. Uncropped and unprocessed images of chromatogram are included in the Source data file. Source data are provided with this paper.

## Source data

Source Data
