# Supplementary material for: BST1 regulates nicotinamide riboside metabolism via its glycohydrolase and base-exchange activities
Source: Nat Commun. 2021 Nov 19;12:6767. doi: 10.1038/s41467-021-27080-3 (PMC8604996; doi:10.1038/s41467-021-27080-3)
Supplement: Supplementary file 1 — Supplementary Information [file 41467_2021_27080_MOESM1_ESM.pdf]

Supplementary Information

BST1 regulates nicotinamide riboside metabolism via its glycohydrolase and base-exchange activities

Keisuke Yaku, Sailesh Palikhe, Hironori Izumi, Tomoyuki Yoshida, Keisuke Hikosaka,
Faisal Hayat, Mariam Karim, Tooba Iqbal, Yasuhito Nitta, Atsushi Sato,
Marie E Migaud, Katsuhiko Ishihara, Hisashi Mori, Takashi Nakagawa

Supplementary Figure 1
Supplementary Figure 2
Supplementary Figure 3
Supplementary Figure 4
Supplementary Figure 5
Supplementary Figure 6
Supplementary Method
Supplementary References

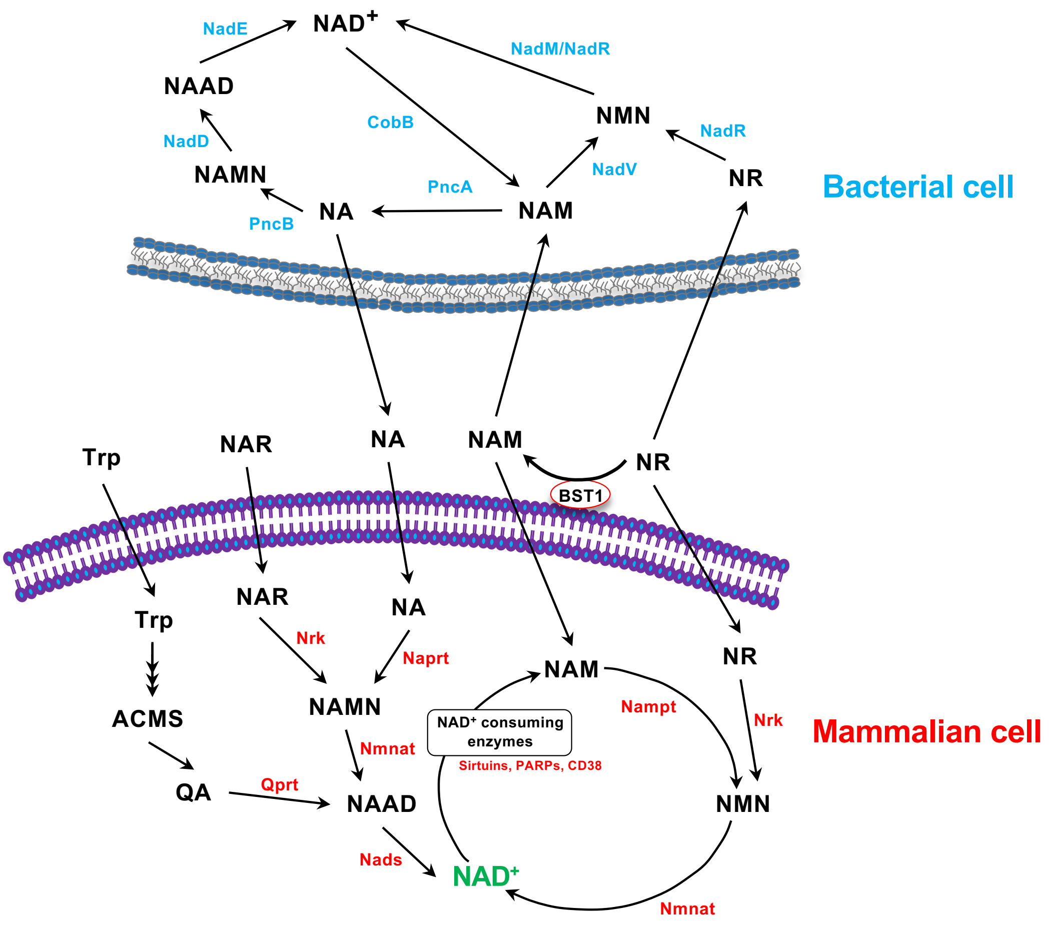

Supplementary Figure 1. Schematic overview of NAD$^+$ biosynthesis pathways in bacterial and mammalian cell.

In mammalian cells, NAD$^+$ can be synthesized from tryptophan (Trp), NAM, NA, NAR and NR. NR is utilized by two different pathways in mammalian cells: direct and indirect pathways. In the direct pathway, NR is taken up by mammalian cells and converted to NMN by Nrk and subsequently to NAD$^+$ by Nmnat. In the indirect pathway, NR is degraded by BST1 extracellularly to produce NAM, which is either taken up by mammalian cells to synthesize NAD$^+$ by the salvage pathway or taken up by bacteria to produce NA catalyzed by PncA. Thus produced NA is utilized by bacteria to synthesize NAD$^+$ as well as released extracellularly which is taken up by mammalian cells to synthesize NAD$^+$ by the Preiss-Handler pathway. NAD$^+$ consuming enzymes such as sirtuins, PARPs, and CD38 produces NAM, which can be recycled to make NAD$^+$.

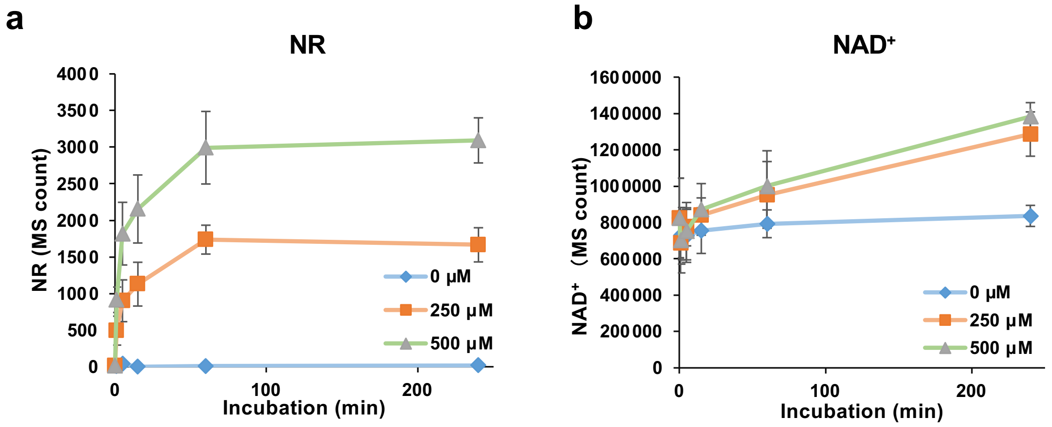

Supplementary Figure 2. NR is directly taken up by A549 cells.

A549 cells were treated with NR (250 μM and 500 μM) and harvested at indicated time points: 0, 1, 5, 15, 60 and 240 min. Relative abundance of NR **(a)** and NAD^+ **(b)** in cells were measured by LC/MS. (*n* = 3 independent experiments) Data are shown as mean ± S.D. Source data are provided as a Source Data file.

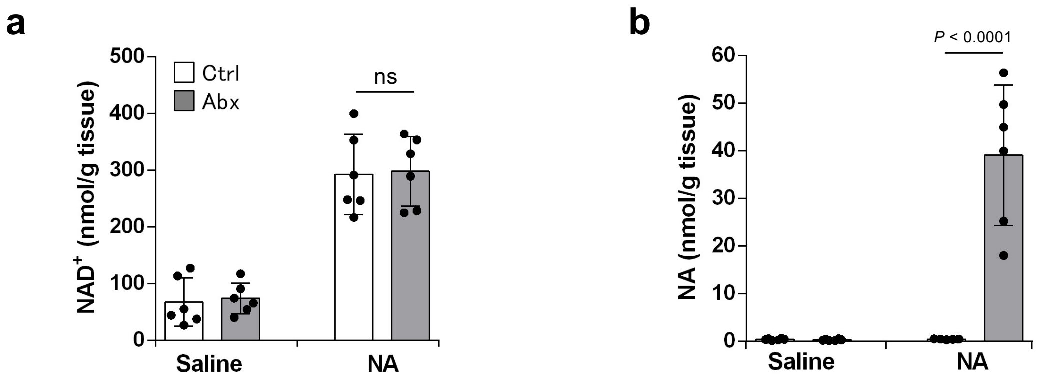

Supplementary Figure 3. Antibiotics treatment could not inhibit the increase of NAD$^+$ levels in the liver at the late phase by oral administration of NA.

Wild type mice were treated with either regular water (Ctrl) or antibiotic-containing water (Abx) for 3 days to deplete gut microbiota. Then, they were gavaged with 193.20 mg/kg NA (equivalent in molar to 400 mg/kg NR), and sacrificed after 3 h of the gavage. **(a, b)** Concentrations of NAD$^+$ **(a,** n = 6 mice per group**)** and NA **(b,** n = 6 mice per group**)** in the liver were measured by LC/MS 3 h after the gavage of NA. Data are shown as mean ± S.D. ns: not significant. Statistical significance was determined by One-way ANOVA followed by Tukey's post-hoc tests. Source data are provided as a Source Data file.

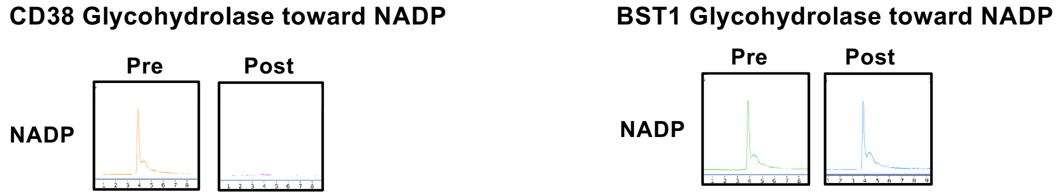

Supplementary Figure 4. CD38, but not BST1, exhibited the glycohydrolase activity against NADP *in vitro*.

NADP was incubated with recombinant **(a)** CD38 or **(b)** BST1 enzymes for 30 min at room temperature. Then, the mixture was subjected to LC/MS analysis for the detection of NADP. Representative chromatograms before and after the incubation were shown (Three independent experiments were performed). Source data are provided as a Source Data file.

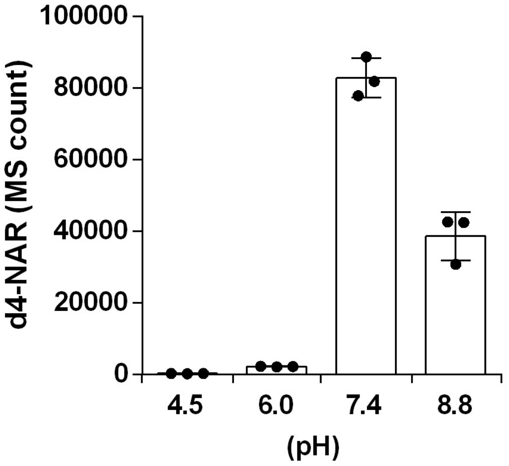

Supplementary Figure 5. BST1 exhibited the most robust base-exchange activity at neutral pH.

Non-labeled NR and d4-NA were incubated with recombinant BST1 protein for 30 min at different pH conditions. Then, the mixture was subjected to LC/MS analysis for the detection of d4-NAR. (*n* = 3 independent experiments)Data are shown as mean ± S.D. Source data are provided as a Source Data file.

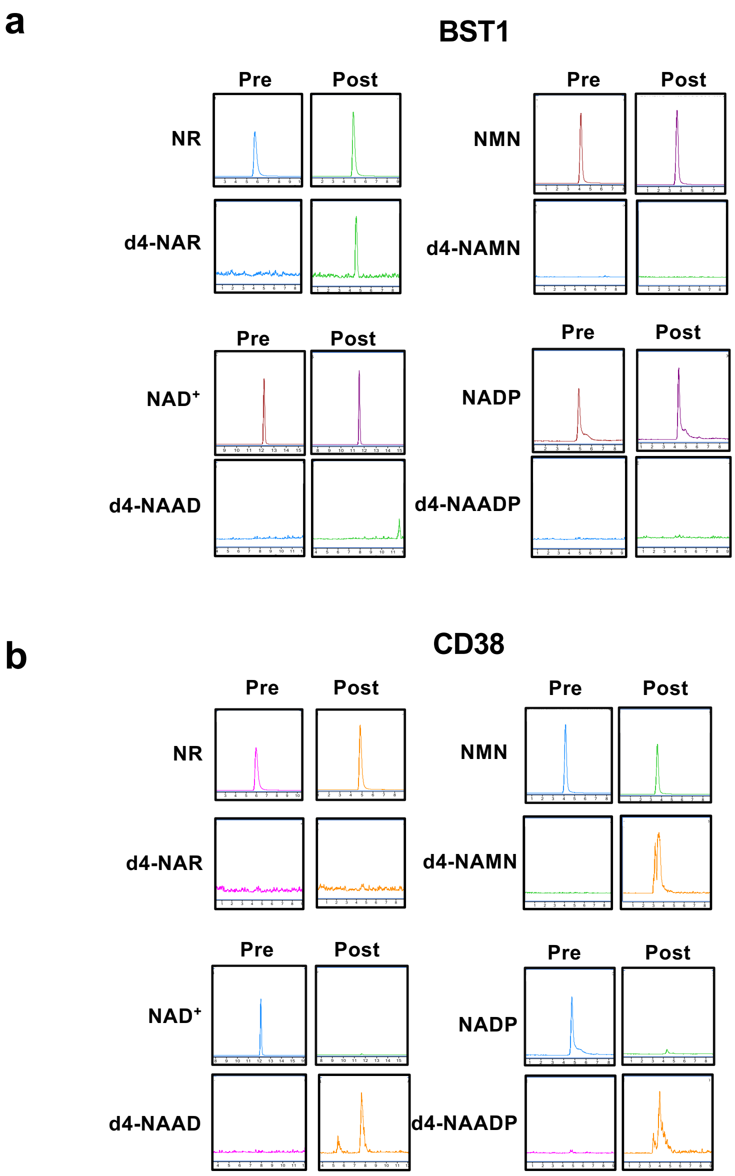

Supplementary Figure 6. CD38, but not BST1, catalyzed the base-exchange reactions against NAMN, NAAD, and NAADP only in the acidic condition.

d4-NA and non-labeled NR, NMN, NAD^+ or NADP were incubated with recombinant BST1 **(a)** or CD38 **(b)** protein for 30 min at room temperature at pH 4.0. Then, the mixture was subjected to LC/MS analysis for the detections of d4-NAR, d4-NAMN, d4-NAAD or d4-NAADP (Three independent experiments were performed). Source data are provided as a Source Data file.

Supplementary Method

Synthesis of quadruple-labeled [18O18O15N13C] NR

Scheme1: Synthesis of ^{13}C, ^{18}O and ^{15}N labelled M+4 nicotinamide

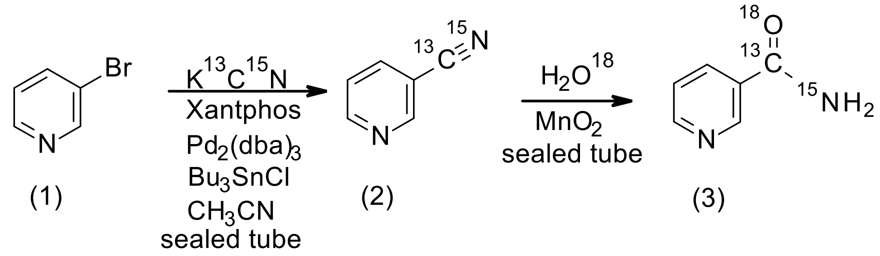

Scheme2: Synthesis of ^{18}O labelled M+4 *p*-nitrobenzoic acid

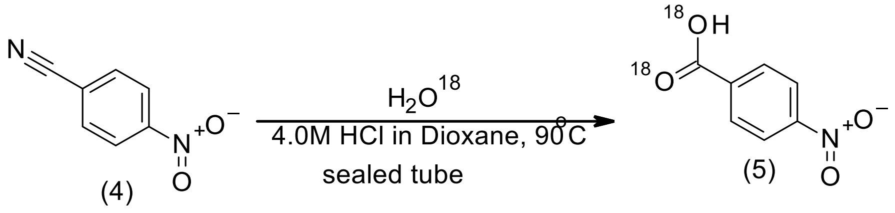

Scheme3: Synthesis of 1,2,3-tri-O-acetyl-β-D-ribofuranoside

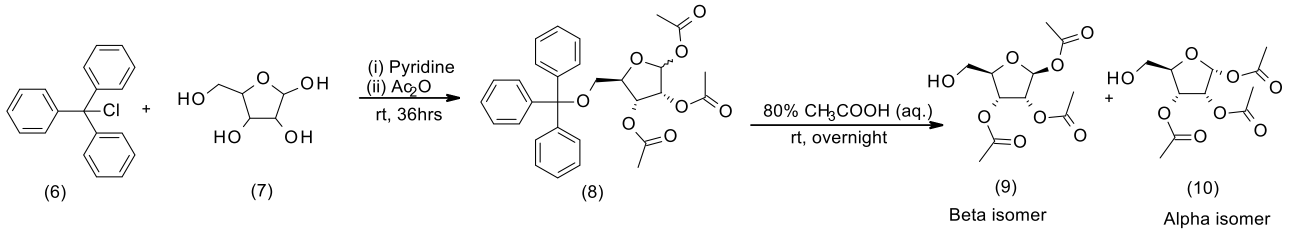

Scheme4: Synthesis of M+6 nicotinamide riboside

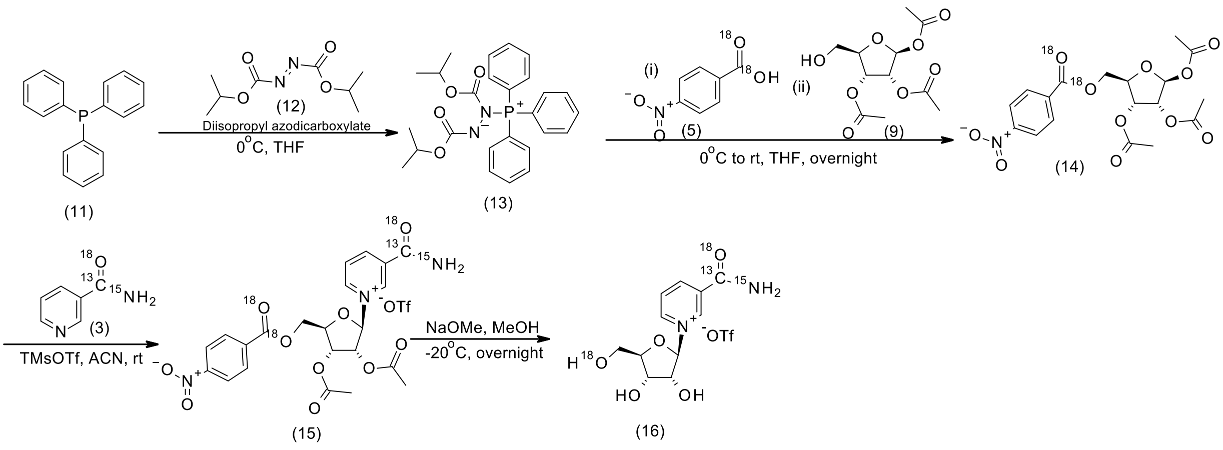

[A] Synthesis of ^{13}C, ^{18}O and ^{15}N labelled M+4 nicotinamide (Scheme 1) : Compound (3) was prepared according to reported procedure [1]

[B] Synthesis of ^{18}O labelled M+4 *p*-nitrobenzoic acid (Scheme 2): Compound (5) was prepared according to reported procedure [2]

[C] Synthesis of 1,2,3-tri-O-acetyl-β-D-ribofuranose (Scheme 3): Compound (9) was prepared according to reported procedure [3]

[D]General procedure for the synthesis of M+6 nicotinamide riboside

Synthesis of M+4 O^{18}labelled β-D-ribose-1,2,3-triacetae-5-p-nitrobenzoate (*compound 14, Step-1&2*): Ph_3P (0.97g, 3.71 mmol, 1.5 eqv) was dissolved in extra dry THF (5 mL). The mixture was then cooled to 0°C under dry nitrogen atmosphere and DEAD (0.721mL, 3.71 mmol, 1.5 eqv) was added. The resulting mixture was stirred until the light-yellow betaine intermediate fully precipitated. ^{18}O Labelled M+4 *p*-nitrobenzoic acid (0.424g, 2.46 mmol, 1.0 eqv) and 1,2,3-tri-O-acetyl-β-D-ribofuranose (0.681g, 2.46 mmol, 1.0 eqv), dissolved in dried THF were the added to the betaine solution. The resulting mixture was stirred at room temperature overnight under inert atmosphere. Upon completion of the reaction, the solid triphenyl phosphonium oxide was filtered off and the solvent was evaporated under reduced pressure. The crude product was used in the next step without purification.

Synthesis of M+6 NR 2,3-diacetate-5-p-nitrobenzoate (*Vorbruggen glycosylation, compound 15, Step-3*): In a flame dried flask under an argon atmosphere, M+4 NAM (0.09g, 0.70 mmol, 1eqv) was added to dry acetonitrile (4mL) at 0 °C. After 5 minutes, TMSOTf (0.63 mL, 3.48 mmol, 5eqv) was added to the stirred acetonitrile solution at the same temperature. The resulting mixture was stirred at rt until complete disappearance of the starting material. Then, a solution of M+4 O^{18}labelled β-D-ribose-1,2,3-triacetae-5-nitrobenzoate (0.300 g, 0.697 mmol, 1eqv) in 2 mL of acetonitrile was added and stirred further for 2h. The reaction was monitored by ^1H NMR analysis of the crude mixture. Upon completion, the resulting solution was concentrated, and the oily crude product was dissolved in anhydrous methanol. Solid $NaHCO_3$ in suspension in methanol was added in a portion-wise manner at 0°C and stirred until the neutralization of remaining TMSOTf was achieved. Upon complete neutralization (pH = 6–7), residual solids were filtered off, and the filtrate was concentrated under reduced pressure. The crude product was used in the next step without purification.

Synthesis of M+6 NR (*de-esterification, compound 16, step-4,*): The crude M+6 NR 2,3-diacetate-5-*p*-nitrobenzoate (0.345g, 0.697 mmol, 1eqv) was dissolved in 2 mL anhydrous methanol and stirred at -20°C. After 15 min of stirring at the same temperature, 0.141mL (312 mM) of a methanolic solution of NaOMe (25% w/v) was added and the resulting mixture was kept in -20°C refrigerator for overnight. The reaction progress was monitored by NMR analysis of crude in acetone-*d6*. Upon completion of the reaction, the reaction mixture was quenched by addition of concentrated acetic acid in a dropwise manner at -80°C with stirring. Upon complete neutralization of excess NaOMe (pH = 6–7), residual solids were filtered off, and the filtrate was concentrated under reduced pressure and the crude product was dissolved in water, adsorbed on silica and purified by C_{18} column chromatography through gradient elution by using methanol : water (2%:98% to 20%:80%) as eluents. Yield 38%, ^{1}H NMR (400 MHz, D2O, δ, ppm): 9.50 (s, 1H, Ar-H), 9.16 (d, J = 5.92 Hz, 1H, Ar-H), 8.88 (d, J = 4.64 Hz, 1H, Ar-H), 8.17 (t, J = 7.0 Hz, 1H, Ar-H), 6.14 (d, J = 4.04 Hz, 1H, H-1), 4.42-4.37 (m, 2H, H_2&H_4), 4.25 (t, J = 4.38 Hz, 1H, H-3), 3.95-3.79 (AB part of ABX system, 2H, J_{AB}=12.8 Hz, J_{AX}=2.04 Hz, J_{BX}=3.04Hz, H-5_{AB}); ^{13}C NMR (100 MHz, D_2O, δ, ppm): 170.73 (d, J=17.6Hz,CO), 145.61 (C-6_{NAM}), 142.58 (C-2_{NAM}), 140.34 (C-4_{NAM}), 133.31 (d, J= 56.4Hz, C-3_{NAM}), 128.33 (C-5_{NAM}), 119.57 (CF_3, d, J_{CF}= 119.5Hz), 99.89 (C-1_{ribose}), 87.66 (C-4_{ribose}), 77.41 (C-2_{ribose}), 69.74 (C-3_{ribose}), 60.13 (C-5_{ribose}); 19F NMR (377 MHz, D_2O) δF: −78.85 (s); HRMS calcd for $C_{10}C^{13}H_{15}N^{15}NO_3(O^{18})_2$ $[M]^+$ 261.1070 found 261.1051.

Supplementary References

[1] Shats I, Williams JG, Liu J, Makarov MV, Wu X, Lih FB, Deterding LJ, Lim C, Xu X, Randall TA, Lee E, Li W, Fan W, Li JL, Sokolsky M, Kabanov AV, Li L, Migaud ME, Locasale JW, Li X. Bacteria boost mammalian host NAD metabolism by engaging the deamidated biosynthesis pathway, Cell Metab. 31 (3), 564-579. e7. (2020).

[2] Beddoe RH, Edwards DC, Goodman L, Sneddon HF, Denton RM. Synthesis of 18O-labelled alcohols from unlabelled alcohols. Chem Commun. 56, 6480-3. (2020).

[3] Beigelman LN, Mikhailov SN. Transient protection in nucleoside synthesis using trityl groups: is it necessary to block hydroxyl groups? Carbohydrate Research, 203(2), 324-9. (1990)

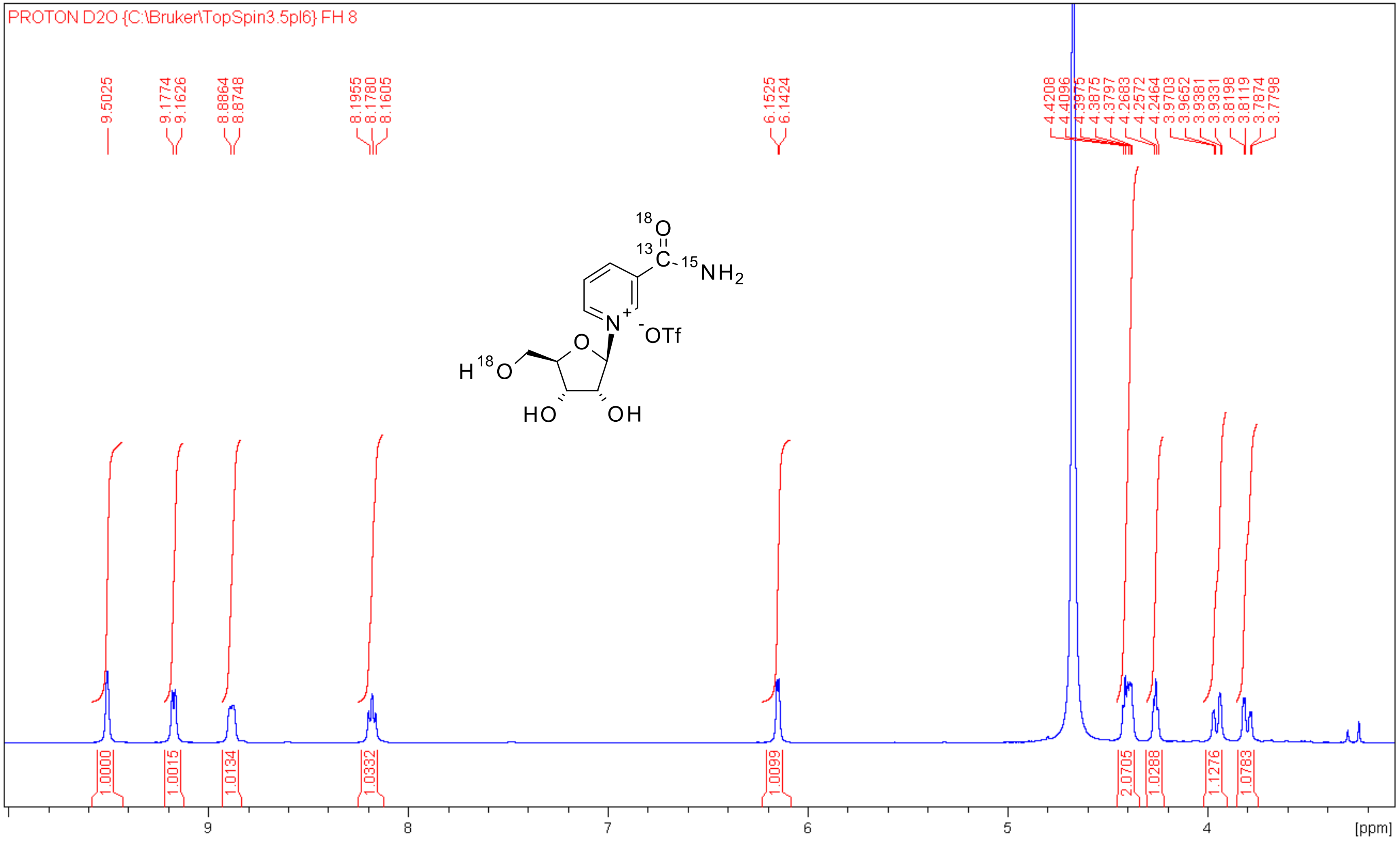
PROTON D2O {C:\Bruker\TopSpin3.5pl6} FH 8
9.5025
9.1774
9.1626
8.8864
8.8748
8.1955
8.1780
8.1605
6.1525
6.1424
4.4208
4.4096
4.3975
4.3875
4.3797
4.2683
4.2572
4.2464
3.9703
3.9652
3.9381
3.9331
3.8198
3.8119
3.7874
3.7798
18O
13C
15NH2
N+
-OTf
O
H 18O
HO
OH
1.0000
1.0015
1.0134
1.0332
1.0099
2.0705
1.0288
1.1276
1.0783
9
8
7
6
5
4
[ppm]

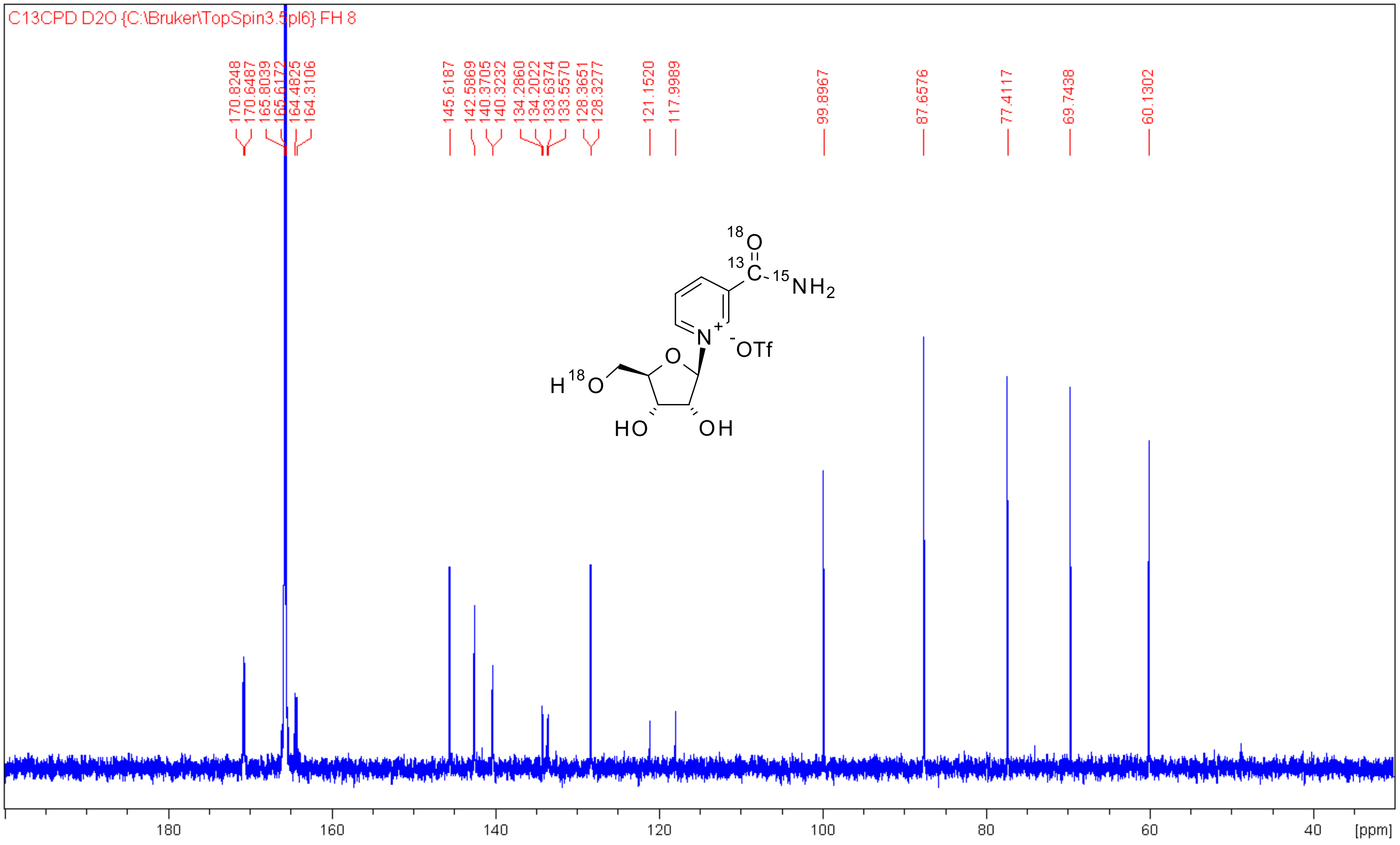

C13CPD D2O {C:\Bruker\TopSpin3.5pl6} FH 8
170.8248
170.6487
165.8039
165.6172
164.4825
164.3106
145.6187
142.5869
140.3705
140.3232
134.2860
134.2022
133.6374
133.5570
128.3651
128.3277
121.1520
117.9989
99.8967
87.6576
77.4117
69.7438
60.1302
18
O
13
C
15
NH2
N
+
-
OTf
O
H
18
O
HO
OH
180
160
140
120
100
80
60
40
[ppm]

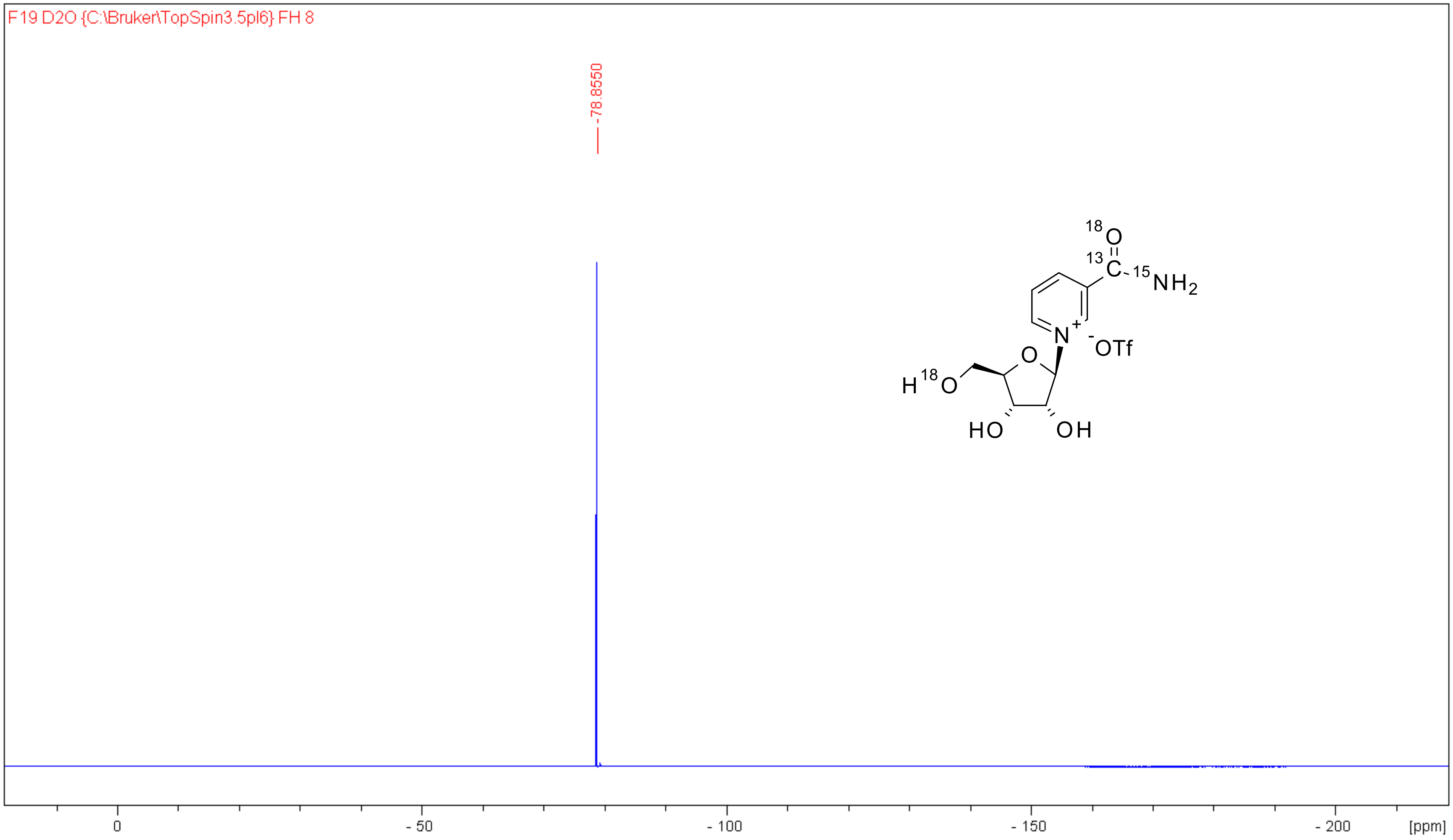
F19 D2O {C:\Bruker\TopSpin3.5pl6} FH 8
-78.8550
0
- 50
- 100
- 150
- 200
[ppm]
18
O
13
C
15
NH2
N
+
-
OTf
O
H
18
O
HO
OH

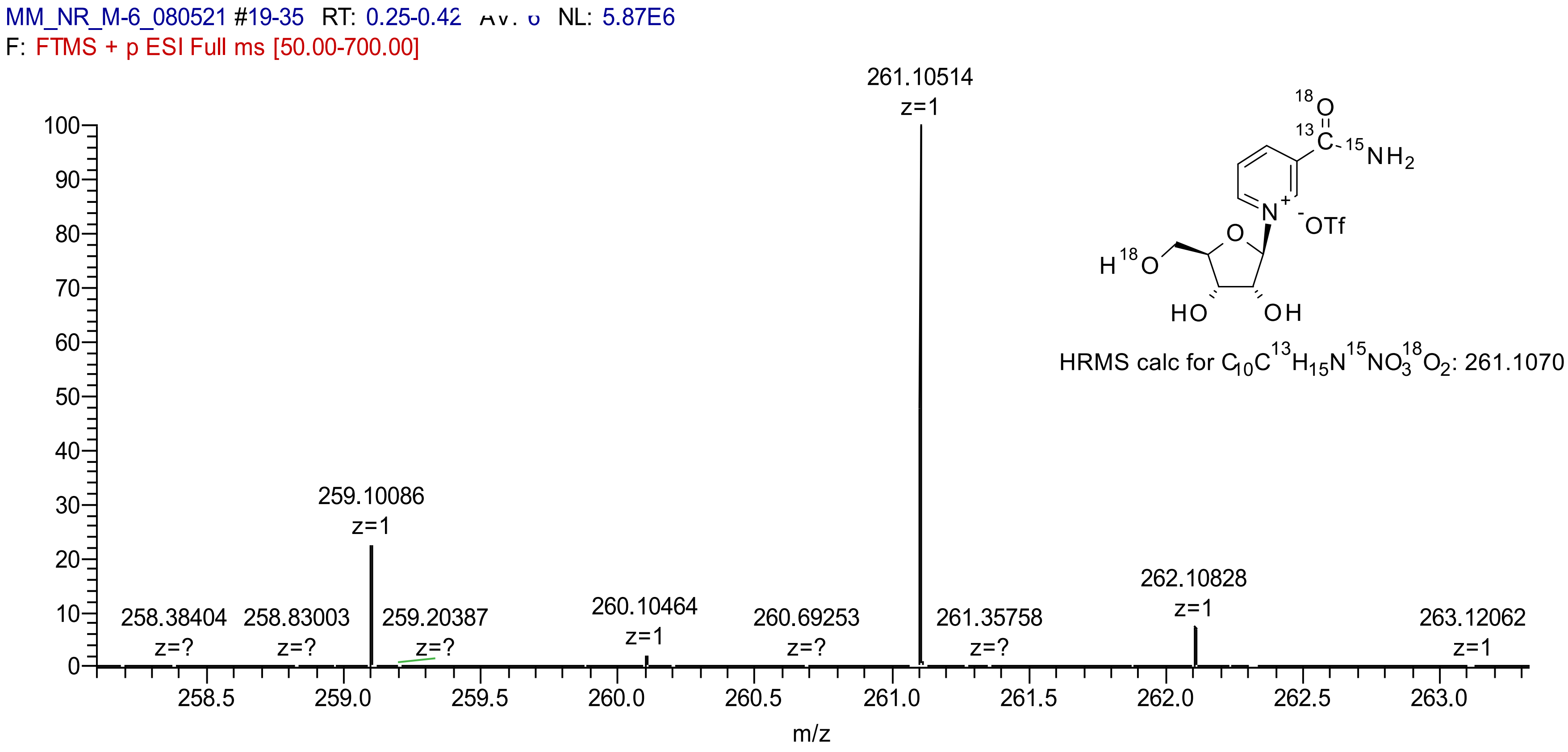

HRMS calc for $C_{10}C^{13}H_{15}N^{15}NO_3^{18}O_2$: 261.1070

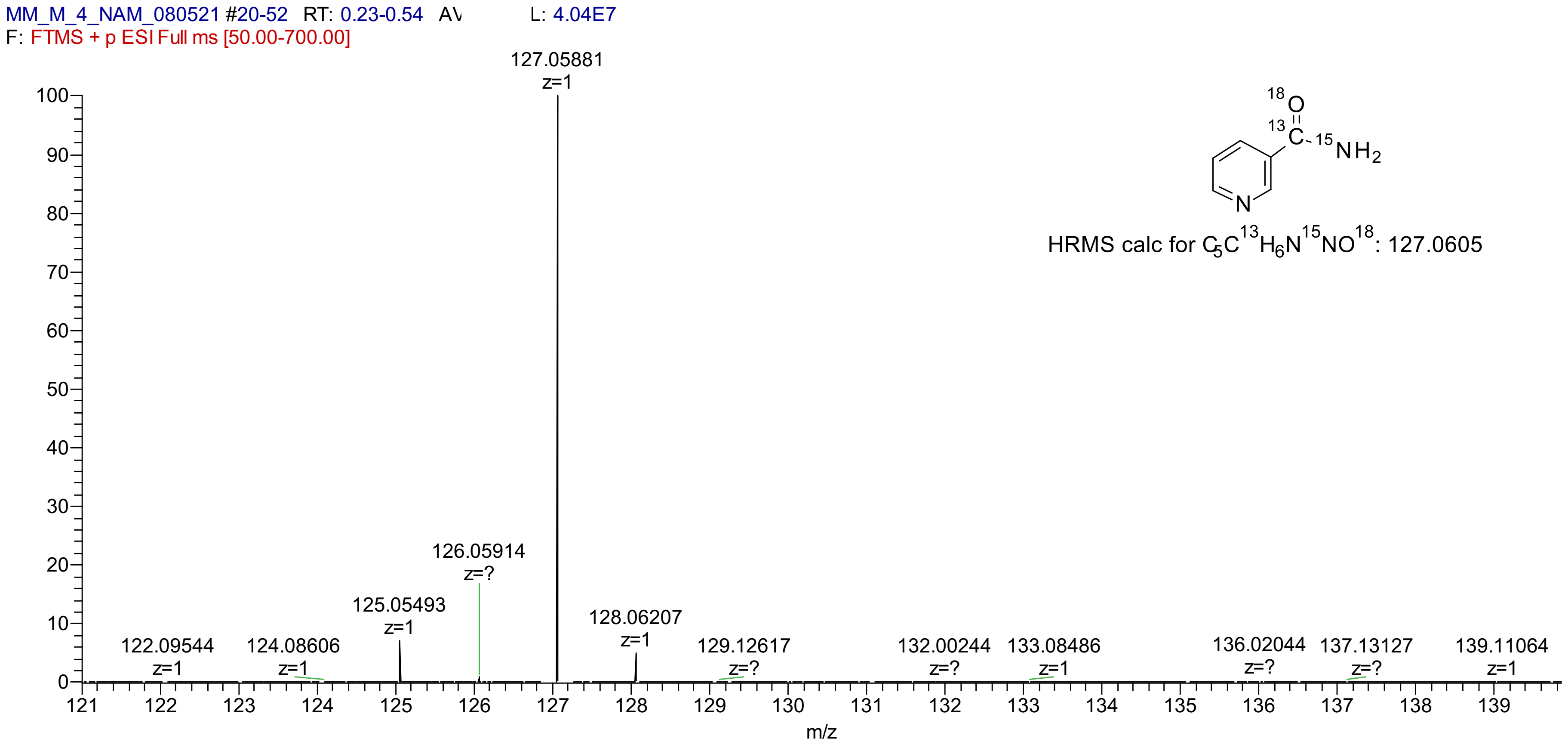
MM_M_4_NAM_080521 #20-52 RT: 0.23-0.54 AV L: 4.04E7
F: FTMS + p ESI Full ms [50.00-700.00]
127.05881 z=1
126.05914 z=?
125.05493 z=1
128.06207 z=1
122.09544 z=1
124.08606 z=1
129.12617 z=?
132.00244 z=?
133.08486 z=1
136.02044 z=?
137.13127 z=?
139.11064 z=1
m/z
HRMS calc for C5C13H6N15NO18: 127.0605
