# Supplementary material for: BST1 regulates nicotinamide riboside metabolism via its glycohydrolase and base-exchange activities
Source: Nat Commun. 2021 Nov 19;12:6767. doi: 10.1038/s41467-021-27080-3 (PMC8604996; doi:10.1038/s41467-021-27080-3)
Supplement: Supplementary file 2 — Reporting Summary [file 41467_2021_27080_MOESM2_ESM.pdf]

Corresponding author(s): Takashi Nakagawa
Last updated by author(s): Sep 29, 2021

Reporting Summary

Nature Portfolio wishes to improve the reproducibility of the work that we publish. This form provides structure for consistency and transparency in reporting. For further information on Nature Portfolio policies, see our Editorial Policies and the Editorial Policy Checklist.

Statistics

For all statistical analyses, confirm that the following items are present in the figure legend, table legend, main text, or Methods section.

n/a	Confirmed	
☐	☒	The exact sample size (*n*) for each experimental group/condition, given as a discrete number and unit of measurement
☐	☒	A statement on whether measurements were taken from distinct samples or whether the same sample was measured repeatedly
☐	☒	The statistical test(s) used AND whether they are one- or two-sided<br>*Only common tests should be described solely by name; describe more complex techniques in the Methods section.*
☒	☐	A description of all covariates tested
☐	☒	A description of any assumptions or corrections, such as tests of normality and adjustment for multiple comparisons
☐	☒	A full description of the statistical parameters including central tendency (e.g. means) or other basic estimates (e.g. regression coefficient) AND variation (e.g. standard deviation) or associated estimates of uncertainty (e.g. confidence intervals)
☐	☒	For null hypothesis testing, the test statistic (e.g. *F*, *t*, *r*) with confidence intervals, effect sizes, degrees of freedom and *P* value noted<br>*Give P values as exact values whenever suitable.*
☒	☐	For Bayesian analysis, information on the choice of priors and Markov chain Monte Carlo settings
☒	☐	For hierarchical and complex designs, identification of the appropriate level for tests and full reporting of outcomes
☒	☐	Estimates of effect sizes (e.g. Cohen's *d*, Pearson's *r*), indicating how they were calculated

Our web collection on statistics for biologists contains articles on many of the points above.

Software and code

Policy information about availability of computer code

Data collection	MassHunter Workstation-Data Acquisition (Version B.05.00, Agilent technologies, Santa Clara, CA, USA) was used to measure samples for metabolomics.
Data analysis	MassHunter Workstation-Quantitative Analysis (Version B.05.00, Agilent technologies, Santa Clara, CA, USA) was used to quantify metabolites.<br>MassHunter Workstation-Qualitative Analysis (Version B.05.00, Agilent technologies, Santa Clara, CA, USA) was used to obtain images of chromatograms.<br>GraphPad Prism 9 (Version 9.1.2, GraphPad Software, San Diego, CA, USA) was used to obtain nonlinear Michaelis-Menten regression and to calculate values of Vmax and Km.<br>Statistical analysis was performed by using GraphPad Prism 9 (Version 9.1.2, GraphPad Software, San Diego, CA, USA).

For manuscripts utilizing custom algorithms or software that are central to the research but not yet described in published literature, software must be made available to editors and reviewers. We strongly encourage code deposition in a community repository (e.g. GitHub). See the Nature Portfolio guidelines for submitting code & software for further information.

Data

Policy information about availability of data

All manuscripts must include a data availability statement. This statement should provide the following information, where applicable:

- Accession codes, unique identifiers, or web links for publicly available datasets
- A description of any restrictions on data availability
- For clinical datasets or third party data, please ensure that the statement adheres to our policy

All the data related to the findings of this work is available on the paper and its Supplementary information. Source data file provides individual data including uncropped images. We don't have the data relevant to Mandates for specific datasets in editorial policy.

Field-specific reporting

Please select the one below that is the best fit for your research. If you are not sure, read the appropriate sections before making your selection.

☒ Life sciences ☐ Behavioural & social sciences ☐ Ecological, evolutionary & environmental sciences

For a reference copy of the document with all sections, see nature.com/documents/nr-reporting-summary-flat.pdf

Life sciences study design

All studies must disclose on these points even when the disclosure is negative.

Sample size	Sample size of each experiment is indicated in the figure legends. Sample size was determined from preliminary experiment. Additionally, publications from other groups suggested that 3-6 animals are enough to obtain statistically significant results in gavage experiments (Trammell et al., Nat. Commun. 2016, doi: 10.1038/ncomms12948 (2016), Shats et al., Cell Metab. 2020, doi.org/10.1016/j.cmet.2020.02.001).<br>Due to limited availability of stable-isotope labeled NAD+ precursors and KO mice, less sample size was used in Fig. 3, Fig. 6, Fig. 8, and Fig. 9.
Data exclusions	To exclude extreme outliers, Smirnov-Grubbs test was performed before analysis based on p-values less than 0.01 in Fig. 2b. Data excluded was also shown in source data.
Replication	The numbers of replicates in each experiment are described in each figure legends. For animal experiments, 3-6 animals per group were used to confirm biological reproducibility. Three distinct cultured cells were used for cell experiments. Technical triplicates were used for in vitro enzymatic assay. All the replications tested were successful.
Randomization	Animals were randomly assigned for each test group. For cell experiments, cells were randomly assigned in each culture.
Blinding	Experiments were not blinded because our work was based on basic biochemical assay and investigators were needed to distinguish controls and other groups.

Reporting for specific materials, systems and methods

We require information from authors about some types of materials, experimental systems and methods used in many studies. Here, indicate whether each material, system or method listed is relevant to your study. If you are not sure if a list item applies to your research, read the appropriate section before selecting a response.

Materials & experimental systems

n/a	Involved in the study	
☒	☐	Antibodies
☐	☒	Eukaryotic cell lines
☒	☐	Palaeontology and archaeology
☐	☒	Animals and other organisms
☒	☐	Human research participants
☒	☐	Clinical data
☒	☐	Dual use research of concern

Methods

n/a	Involved in the study	
☒	☐	ChIP-seq
☒	☐	Flow cytometry
☒	☐	MRI-based neuroimaging

Eukaryotic cell lines

Policy information about cell lines

Cell line source(s)	Caco-2 and A549 cells were obtained from Riken BRC (Japan).

Authentication	The cells were authenticated by Riken BRC (Japan).
Mycoplasma contamination	Mycoplasma free cells were provided from the supplier.
Commonly misidentified lines (See ICLAC register)	No commonly misidentified cell lines were used in our study.

Animals and other organisms

Policy information about studies involving animals; ARRIVE guidelines recommended for reporting animal research

Laboratory animals	Eight to ten weeks old male mice were used in this study.<br>C57BL/6N mice were obtained from Japan SLC Inc. (Shizuoka, Japan). BST1 knockout mice were obtained from RIKEN BRC (Stock No. RBRC02401). CD38 knockout mice were obtained from RIKEN BRC (Stock No. RBRC01462). NADS knockout mice were obtained from International Mouse Phenotyping Consortium (MMRRC:048728-UCD). Naprt knockout mice were generated using CRISPR-Cas9 techniques.<br>Animals were kept under a controlled temperature around 25°C with humidity kept around 50%.
Wild animals	No wild animals were used in this study.
Field-collected samples	No field-collected samples were used in this study.
Ethics oversight	All the animal experiments were approved by the Animal Experiment Committee at the University of Toyama and were performed in accordance with the Guidelines for the Care and Use of Laboratory Animals at the University of Toyama, which are based on international policies. All relevant ethical regulations have been complied.

Note that full information on the approval of the study protocol must also be provided in the manuscript.
